# Optimal testing frequency for sexually transmitted infections among men who have sex with men and transgender women who use HIV pre-exposure prophylaxis in Australia, Brazil and Thailand: a cost-effectiveness analysis

**DOI:** 10.1016/j.lanwpc.2026.101837

**Published:** 2026-04-01

**Authors:** Rui Zhao, Maeve Brito de Mello, Pâmela Cristina Gaspar, Angelica Espinosa Miranda, Nittaya Phanuphak, Philippe Mayaud, Angela Carvalho Freitas, Katia Cristina Bassichetto, Maria Amelia Veras, Daniel McCartney, Jiajun Sun, Hao Lai, Filip Meheus, Jason J. Ong, Lei Zhang

**Affiliations:** a Shenzhen Hospital of Southern Medical University, Shenzhen, Guangdong, China; b Global HIV, Hepatitis and STI Programmes, World Health Organization, Geneva, Switzerland; c Departamento de HIV/AIDS, Tuberculose, Hepatites Virais e Infecçoes Sexualmente Transmissíveis, Secretaria de Vigilância em Saúde e Ambiente, Ministério da Saúde, Brasilia, Distrito Federal, Brazil; d Departamento de Medicina Social, Universidade Federal do Espírito Santo, Vitória, Espírito Santo, Brazil; e Institute of HIV Research and Innovation, 319 Phayathai Road, Pathumwan, Bangkok, 10330, Thailand; f Center of Excellence in Transgender Health, Faculty of Medicine, Chulalongkorn University, 1873 Rama IV Road, Pathumwan, Bangkok, 10330, Thailand; g Clinical Research Department, London School of Hygiene & Tropical Medicine, London, United Kingdom; h Departamento de Infectologia e Medicina Tropical, Hospital das Clinicas HCFMUSP, Faculdade de Medicina, Universidade de Sao Paulo, Sao Paulo, SP, Brazil; i Faculty of Medical Sciences of Santa Casa de São Paulo, Rua Dr Cesario Mota Jr 61, São Paulo, SP, 01221-020, Brazil; j Faculdade de Ciências Médicas da Santa Casa de São Paulo, Rua Dr Cesario Mota Jr 61, São Paulo, SP, 01221-020, Brazil; k Phase I Clinical Trial Research Ward, The Second Affiliated Hospital of Xi'an Jiaotong University, No.157 Xi Wu Road, Xi'an, 710004, Shaanxi Province, PR China; l China-Australia Joint Research Center for Infectious Diseases, School of Public Health, Xi'an Jiaotong University Health Science Center, Xi'an, Shaanxi, 710061, PR China; m Department of Performance, Financing and Delivery. World Health Organization. Geneva, Switzerland; n Faculty of Infectious and Tropical Diseases, London School of Hygiene and Tropical Medicine, London, United Kingdom; o Melbourne Sexual Health Centre, Alfred Health, Melbourne, VIC, Australia; p School of Translational Medicine, Faculty of Medicine, Nursing and Health Sciences, Monash University, Melbourne, VIC, Australia

**Keywords:** Sexually transmitted infection, Men who have sex with men, Transgender women, Pre-exposure prophylaxis, Cost-effectiveness

## Abstract

**Background:**

Pre-exposure prophylaxis (PrEP) is highly effective in preventing HIV, but not other sexually transmitted infections (STIs) in men who have sex with men (MSM) and transgender women (TGW). PrEP users are offered periodic STI testing for early detection of STIs, including *Chlamydia trachomatis* (CT), *Neisseria gonorrhoeae* (NG) and syphilis. However, the optimal and most cost-effective frequency for separate and combined STI testing in PrEP users has yet to be established.

**Methods:**

We constructed dynamic transmission models and simulated the epidemic trajectories of three STIs (CT, NG and syphilis) among MSM/TGW using PrEP in Australia, Brazil and Thailand. Two testing scenarios were considered: single pathogen testing for CT, NG or syphilis, and combined testing for all three STIs simultaneously (syphilis testing alone plus dual testing for CT/NG). From a healthcare system perspective, cost-effectiveness analysis and incremental cost-effectiveness ratio (ICER) was used to explore the optimal testing strategy for STIs across three countries over a 5-year time horizon.

**Findings:**

For the single CT/NG testing scenario, either maintaining status quo, or every 6 or 12 months was more cost-effective for Australian MSM, Brazilian MSM and Thai MSM/TGW, while 3-monthly CT/NG testing was optimal for Brazilian TGW. For the single syphilis testing scenario, 3-monthly testing was more cost-effective for Australian, Brazilian MSM/TGW and Thai TGW, while 6-monthly testing was optimal for Thai MSM. For the combined testing strategy, 3-monthly dual testing for CT/NG, along with 3-monthly syphilis testing for Australian MSM and Brazilian TGW, was a cost-saving and optimal strategy, but exceeded the willingness-to-pay threshold for Brazilian MSM (ICER = $23,187/QALY gained), Thai MSM (ICER = $477,442/QALY gained) and Thai TGW (ICER = $37,697/QALY gained).

**Interpretation:**

Our study suggests that more frequent (3-monthly) syphilis testing for PrEP users provides enhanced economic value among both MSM and TGW. However, less frequent CT and NG testing may only be required, depending on the background disease burden and the costs associated with testing and management in each country.

**Funding:**

Supported by the 10.13039/100004423World Health Organization (Grant Number: INV-035239).

**Research in context**

**Evidence before this study**

Pre-exposure prophylaxis (PrEP) is a safe and effective HIV prevention strategy for key populations like men who have sex with men (MSM) and transgender women (TGW). However, as its use might be linked to more condomless sex or sexual partners, regular testing for sexually transmitted infections (STIs) among PrEP users is vital to detect STIs early and curb their spread. Although the WHO and some countries recommend frequent STI testing for those at high HIV risk, there's no established optimal testing interval for PrEP users. From a public health perspective, exploring the optimal STI testing frequency with the most cost-effectiveness must be considered, particularly in resource-constrained settings with STI testing budget limits. We searched PubMed, Embase, and Web of Science between Jan 1, 2012, and Dec 30, 2024, with no language restrictions, using the terms “PrEP” or “pre-exposure prophylaxis”, “HIV”, “MSM” or “men who have sex with men” or “gay men” or “TGW” or “transgender women”, “sexually transmitted infections” or “sexually transmitted diseases” or “STI”, “test” and “cost-effectiveness” to identify published economic evaluations on exploring the optimal STI testing frequency for MSM/TGW using PrEP around the world.

We found one study has compared the cost-effectiveness of 3-monthly vs. 6-monthly *Chlamydia trachomatis* (CT) and *Neisseria gonorrhoeae* (NG) testing among MSM using PrEP in the Netherlands, one study has evaluated the cost-effectiveness of implementing single and dual HIV/syphilis testing among key populations in Viet Nam, and one study has modelled the impact of different gonorrhoea testing frequencies in Canadian PrEP users. However, none had evaluated the cost-effectiveness of various pathogens and STI testing frequencies for PrEP users.

**Added value of this study**

The frequency of STI testing should be determined by context specific epidemiology, behavioural patterns, health system structures and costs. To our knowledge, this is the first study to formally evaluate the optimal testing frequency of CT, NG and syphilis among MSM/TGW using PrEP in multiple countries. In this study, we developed models to assess the health impact and cost-effectiveness of various STI testing strategies for CT, NG, and syphilis among MSM and TGW using PrEP in Australia, Brazil, and Thailand. These three countries were considered because they represent the established PrEP-friendly policies in developed and developing countries, respectively. In these countries, PrEP is either free or subsidised, making it more accessible. This existing infrastructure and commitment to PrEP provision create a favourable environment for exploring the integration of PrEP with STI testing strategies. The practical experience and data from these settings can offer valuable, real-world insights, enhancing the feasibility and applicability of such combined strategies in diverse economic contexts. Our results provide insights for decision-makers to select suitable STI testing strategies based on their healthcare resources. Our findings suggest that for MSM, more frequent testing may be warranted for syphilis. Conversely, for TGW, different optimal testing frequencies emerged, emphasizing the need for targeted approaches. These results highlight the necessity of flexible, context-specific STI testing strategies to maximize health outcomes and optimize resource allocation within diverse populations.

**Implications of all the available evidence**

The available evidence highlights the significance of regular STI testing for PrEP users in public health. It emphasizes combining biomedical interventions like PrEP with behavioural monitoring through STI testing for comprehensive sexual health. The findings suggest appropriate STI testing frequencies can enable early STI detection and treatment, curbing transmission and healthcare burden. Compared to prior studies, this research underscores the need to establish optimal testing schedules to enhance PrEP programmes' effectiveness. Future research should focus on validating these results across diverse settings and exploring cost-effective integration of regular STI testing into PrEP delivery systems, particularly in resource-limited environments.

## Introduction

Pre-exposure prophylaxis (PrEP) is safe and effective in reducing the risk of HIV infection.^1^, ^2^, ^3^ By 2024, HIV PrEP had been approved for use in 79 countries worldwide.^4^ Based on the latest statistics from PrEPWatch, high-income countries (HICs) like Australia have over 80,000 PrEP initiations to date, covering 56% of PrEP-eligible individuals, while low-middle-income countries (LMICs) like Brazil and Thailand have nearly 217,000 (15.4% coverage) and 79,000 (31.0% coverage) PrEP initiations, respectively.^5^ Men who have sex with men (MSM), transgender women (TGW) and other members of key populations are at increased risk of acquiring HIV and other sexually transmitted infections (STIs), making them eligible for PrEP use.^6^ However, PrEP does not prevent other STIs such as *Chlamydia trachomatis* (CT), *N. gonorrhoeae* (NG) and syphilis. Prior studies have demonstrated that MSM and TGW who use PrEP have a high prevalence of STIs at baseline and a high incidence while on PrEP.^7^, ^8^, ^9^ Therefore, it is important to evaluate the value of regular STI testing for PrEP users, which could facilitate timely identification of STIs, especially for asymptomatic cases,^10^^,^^11^ and potentially reduce further spread across sexual networks.^12^^,^^13^

With the rapid scale-up of PrEP worldwide, more MSM and TGW are engaging with healthcare systems than ever before, providing a unique opportunity to integrate PrEP services with comprehensive STI testing and other sexual health services, particularly in LMICs where such services are currently limited.^14^ Nonetheless, the current implementation of STI testing services among PrEP users varies across countries.^15^ According to a recently published systematic review, LMICs like Thailand and Brazil, and HICs like Australia recommend 3-monthly STI testing for CT, NG and syphilis, along with triple-site (genital, anal, and pharyngeal) CT/NG testing for MSM using PrEP.^14^^,^^15^ However, while more frequent testing across multiple anatomical sites can identify more STI cases, integrating STI testing into PrEP care can significantly increase costs and complicate service delivery,^16^, ^17^, ^18^, ^19^ and may not lead to a decrease in incidence of the infections.^20^^,^^21^

Therefore, although the World Health Organization (WHO) and several countries recommend more frequent STI testing for individuals at substantial risk of HIV infection,^22^, ^23^, ^24^, ^25^, ^26^ evidence is lacking on the appropriate frequency and optimal interval for offering STI testing to people using PrEP.^7^ From a public health perspective, the effectiveness and cost-effectiveness of optimizing STI testing schedules warrant careful consideration, particularly in resource-limited settings where financial constraints may restrict the delivery of essential health services. In response to these challenges, the WHO commissioned Monash University to develop models simulating the transmission and progression of chlamydia, gonorrhoea, and syphilis among MSM and TGW in Australia, Brazil, and Thailand, informed by country-specific STI prevalence and risk behaviours. Using these models, we conducted a cost-effectiveness analysis to identify optimal testing frequencies and strategies for CT, NG, and syphilis screening among MSM and TGW using PrEP.

## Methods

### Modelling review

We constructed dynamic transmission models for STIs using a susceptible–infectious–recovery (SIR) framework for MSM and TGW, respectively. Separate models were developed for chlamydia (CT), gonorrhea (NG), and syphilis to capture differences in transmission dynamics and disease progression once PrEP users acquire STIs (Figure S1 for CT/NG; Figure S2 for syphilis). Australia, Brazil, and Thailand were selected because of their PrEP-friendly policies in both developed and developing settings, where PrEP is either free or subsidized, thereby representing different stages of PrEP implementation and STI testing services among MSM and TGW.^27^ This infrastructure provides a strong foundation for examining integrated PrEP use-STI testing strategies. In Australia, PrEP use has reached a relatively mature and widely promoted phase, with high awareness and uptake among MSM/TGW. STI testing services are also well established, with broad coverage and accessibility.^28^^,^^29^ In contrast, although Brazil and Thailand have incorporated PrEP into their national health systems or promoted its use through research programs and clinical trials, actual PrEP uptake remains below expected targets.^30^^,^^31^ STI testing coverage among MSM/TGW in these settings also requires improvement, particularly regarding accessibility and utilization.^32^^,^^33^ A key challenge in our modeling work was the absence of reliable estimates for the population size of Australian TGW. Available national data on PrEP prescriptions and HIV incidence do not stratify TGW separately from MSM, limiting our ability to derive essential epidemic and PrEP-use parameters for TGW.^34^^,^^35^ Due to these data constraints—specifically, limited information on TGW population size, PrEP use, and HIV/STI incidence in Australia—we were only able to include Australian MSM in the models.

Each STI model included several health states, ranging from being susceptible to STIs, STIs acquisition, and recovery from STIs either through treatment or natural recovery (Figures S1 and S2). The initial cohort for the CT, NG and syphilis models included MSM and TGW susceptible to HIV infection who either engaged with PrEP programs or not, based on the PrEP coverage in each country. Long-acting injectable PrEP at 2 or 6-month intervals have been approved in many countries and has shown excellent efficacy and safety in several clinical trials.^36^^,^^37^ However, high price and limited coverage of medical insurance, low awareness and acceptance among potential users and healthcare providers, and high reliance on medical facilities and staff impede the scaling-up of long-acting PrEP compared to oral PrEP.^38^^,^^39^ In US, only 1.4% of PrEP users have taken long-acting PrEP after two years of approval in 2021.^40^ Hence, our study considered only oral PrEP users (tenofovir disoproxil fumarate 300 mg/emtricitabine 200 mg) to focus more on exploring how additional STI testing services could be incorporated into current PrEP practices.

As the risk of HIV infection varies over time and depends on the specific risk behaviors of MSM and TGW, individuals suitable for PrEP (i.e., at high risk for HIV infection) may become unsuitable due to a reduction in high-risk behaviors and vice versa. Therefore, in conjunction with current PrEP coverage data for MSM and TGW in the three countries, we defined three susceptible states in our model: a) MSM/TGW suitable and on PrEP; b) MSM/TGW suitable but not on PrEP; and c) MSM and TGW unsuitable and not on PrEP. The rates of sexual mixing among suitable, unsuitable, PrEP users and non-PrEP users were also set for each STI model. In our study, co-infections among the three STIs were not considered. Incorporating co-infections would necessitate additional parameters and data in all three countries to describe the interactions among different STIs as well as their combined effects on health outcomes and costs, which would substantially increase the complexity and uncertainty of the model.

We assumed that individuals who acquired HIV would enter a state with HIV infection and discontinue PrEP use. We also set several health states for PrEP users with or without STIs based on the best available data on the prevalence of CT, NG, and syphilis among MSM and TGW in each country. We assumed that once individuals acquired CT or NG, those with symptomatic infections would seek and accept treatment, while asymptomatic cases would only be identified through STI testing and obtain treatment. Due to the significant differences in the actual risks of long-term effects after STI acquisition among different populations and the limited relevant data, to simplify our model, we did not incorporate long-term and specific consequences of symptomatic and asymptomatic CT/NG infections, such as prostatitis and epididymitis. Diagnosed STI cases would be treated based on country-specific first-line treatment guidance. Given the complexity of drug resistance, insufficient data for modeling, and its relatively small short-term impact on STI testing and treatment, the resistance of NG to first-line recommended medicines was not considered. Individuals with STIs could also experience natural recovery or death, and those recovered individuals after treatment or natural clearance would re-enter the susceptible states (Figures S3 and S4). Overall, we have constructed 8 disease state compartments for CT/NG and 24 compartments for syphilis, respectively.

### Data sources

All model parameters were obtained from published literature, public databases, and in-country collaborators. Health and disease-related parameters, including STI incidence, mortality, transition probabilities between health states, and utility values, were obtained from published sources to simulate the progression and treatment of STIs. Background mortality for each country was obtained from the United Nations Population Division and the World Bank for adult males in 2020.^41^ We estimated population sizes, including those PrEP users with or without STIs in MSM/TGW in Australia, Brazil, and Thailand, respectively. Cost data included the medical costs of PrEP drugs, medical consultations, and laboratory tests related to STI testing and other tests for PrEP monitoring, STI treatment, and personnel costs for all healthcare services involved. To simplify the costing and allow the study to focus on the core objectives of the testing strategy, we assumed that no direct medical costs were assigned to the diagnosis and treatment of asymptomatic CT/NG cases that not identified by STI testing, as they would not seek STI testing and treatment without symptoms. Unit prices for laboratory tests, medical consultations, and STI treatment were obtained from publicly available databases in each country. All costs in the results are presented in the 2024 US$.

Health effects were measured as quality-adjusted life years (QALYs) associated with specific health conditions, calculated using the health utility and duration of each condition in years. The utility values were derived from the Health Utilities Index developed by a committee of the Institute of Medicine (US) in Vaccines for the 21st century.^42^ Due to the limited research data on QALYs related to asymptomatic infections and the fact that we did not consider the specific complications caused by CT/NG infection, we assumed that asymptomatic CT/NG infections would not lead to loss of QALYs due to the absence of symptoms, to make a conservative estimate of the model results (Tables S1–S4).

### Testing scenarios and strategies

The status quo (current STI testing practice) was defined as the representative baseline scenario and served as the benchmark for comparing alternative strategies in our model. The status quo reflected the existing STI testing frequency among MSM/TGW in each country^43^, ^44^, ^45^, ^46^: 3.22 CT/NG tests and 1.8 syphilis tests per year for Australian MSM; 0.2 CT/NG tests and 0.7 syphilis tests per year for Brazilian MSM/TGW; and 0.4 CT/NG/syphilis tests per year for Thai MSM/TGW.

Building on the status quo, we evaluated potentially optimal testing approaches for CT, NG, and syphilis among PrEP users by assuming two testing scenarios for MSM and TGW in each country. First, the single-pathogen testing scenario used one PCR kit for CT and another for NG, based on pooled testing of three anatomical sites (i.e., a single mixed sample using two swabs from the oropharyngeal and anorectal sites and a urine sample), with separate treponemal and non-treponemal serology for syphilis. Second, the combined testing scenario used a dual CT/NG PCR kit for pooled triple-site screening, accompanied by separate serologic testing for syphilis. To simplify the model, we assumed that CT/NG PCR sensitivity was approximately 100%, whereas syphilis test sensitivity varied by stage of infection (e.g., primary vs. secondary syphilis). Test specificity was not incorporated, as the analysis focused primarily on individuals who were truly infected with STIs.

Given the limited resources for STI prevention, designing the optimal testing frequency requires a trade-off between population impact and intervention efficiency. According to the recommendations of US CDC, for MSM at increased risk (such as those using PrEP, infected with HIV, or having multiple sexual partners), the frequency of STI testing can be adjusted every 3–6 months.^47^ Another modelling study demonstrated that 12-monthly HIV/STI testing frequency for PrEP users was the most cost-effective testing strategy.^27^ Therefore, we explored four STI testing frequencies (status quo, every 3, 6 and 12 months) for each testing scenario, resulting in a total of 22 STI testing strategies among MSM and TGW in each country (12 strategies for ‘single pathogen testing’ scenario, and 10 strategies for ‘combined testing’ scenario). For PrEP usage, MSM and TGW suitable for PrEP, whether using PrEP or not, would be followed up for STI testing at one of these three frequencies. Those unsuitable and not on PrEP were assumed to receive STI testing according with the current practices (status quo) in each country. Once an individual acquired HIV, they would be no longer be suitable for PrEP and would be linked to HIV-related treatment and no longer be followed up for our STI testing strategies.

### Cost-effectiveness analysis

Our model evaluates population-level impacts, medical costs, and the cost-effectiveness of various strategies and STI testing frequencies among PrEP users with a monthly cycle length over 5 years. As prolonged simulation horizons may compromise the immediacy of the findings for short-term decision-making, a 5-year modeling cycle offers timely, actionable insights for decision-makers, enabling them to optimize resource allocation for PrEP and STI testing services in the current and upcoming years.^48^^,^^49^ Furthermore, strategies estimated as cost-effective in the short term are likely to become more cost-effective when assessed over longer time horizons.

The incremental cost-effectiveness ratio (ICER), measured as cost per QALY gained, was calculated to compare different strategies and identify the optimal STI testing frequency for PrEP users in each country. Net monetary benefit (NMB, calculated as incremental effectiveness × willingness-to-pay threshold, minus incremental cost) was also applied to measure the optimal STI testing strategy under certain willingness-to-pay thresholds in each country. The strategy with the highest net monetary benefit was defined as the preferred strategy at the specified WTP threshold. The benefit-cost ratio (BCR) was also employed to quantify the monetary return on investment in PrEP and STI testing scenarios that were estimated to be cost-saving. BCR was calculated as the number of dollars saved in direct medical costs of STI treatment for every dollar invested in STI testing: BCR ≥1 indicates that intervention has net economic benefits, while BCR <1 implies that benefits are insufficient to cover the costs. Additional outcome measures included total health system expenditures on PrEP drug and monitoring, STI testing and treatment, the number of new STI cases, the number of diagnosed STI cases, the cost per STI case averted, and the cost per STI case diagnosed by each strategy. The willingness-to-pay (WTP) thresholds were set based on the standards in each country, which were $31,500 (AUD 50,000) for Australia,^50^ $8786 (BRL 505,309) for Brazil,^51^ and $4800 (THB 160,000) for Thailand.^52^^,^^53^ All future costs and QALYs were discounted at an annual rate of 3% in the three countries.^54^

### Sensitivity analysis

In addition to the base-case analysis, one-way and probabilistic sensitivity analyses (PSA) were also performed for each single pathogen testing scenario on key model input parameters and assumptions used. Specifically, one-way sensitivity analyses, presented as tornado diagrams, were performed to assess the impact of uncertainty of the variation of single parameter, focusing on the variation of cost-effectiveness between more frequent (3-monthly) STI testing and status quo (current practice). PSA was conducted using 1000 Monte Carlo simulations to evaluate the combined uncertainty of all model parameters on ICERs.^55^

### Ethics approval

Ethics approval was not required for this study as it was based on modelling research and did not involve human or animal subjects.

### Role of the funding source

The funder of the study had no role in the study design, data collection, data analysis, data interpretation, or report writing.

## Results

### Australia (willingness-to-pay threshold = $31,500)

#### Men who have sex with men

For the single pathogen testing scenario for PrEP users under the current WTP threshold ($31,500), the optimal testing frequencies were maintaining the status quo (testing 3.22 times per year) for CT, every 12 months for NG (ICER = $73,000/QALY gained, BCR = 0.22), and every 3 months for syphilis (cost-saving, BCR = 21.52). Overall, this screening approach would identify 10,246 new CT cases, 5987 new NG cases, and 59,951 new syphilis cases at costs of $29,857, $51,356, and $4760 per CT, NG, and syphilis case diagnosed, respectively. The most cost-effective combined testing strategy (dual CT/NG testing with urine, anorectal, and oropharyngeal swabs, plus separate syphilis testing for PrEP users) was 3-monthly dual testing for CT/NG, along with 3-monthly separate syphilis testing (cost-saving). This strategy would identify 74,756 new STI cases for $10,465 per STI case diagnosed (Table 1).

**Table 1:** Health, economic outcomes and cost-effectiveness of STI testing frequencies for PrEP users among Australian MSM (for 5 years).

Strategy	Cost, $, thousand					Effectiveness				Cost-effectiveness				
	PrEP related	STI testing	STI treatment	Total	Dif. cost	No. STI cases	No. identified STI cases	QALYs	Dif. QALYs	Cost per case averted, $^a^	Cost per case identified, $	ICER ($/QALY gained)^b^	BCR^c^	NMB^d^, per capita
Single testing for CT														
Status quo^e^	41,191	37,453	227,274	305,918	–	14,559	10,246	620,630	–	–	29,857	–	–	192,439
12mCT	46,860	28,735	237,742	313,338	7420	66,151	31,075	620,423	−207	−144	10,083	dominated	/	192,300
6mCT	42,450	32,795	229,943	305,187	−8151	26,041	15,994	620,580	157	64	19,082	cost-saving	0.57	192,431
3mCT	40,929	40,270	226,682	307,880	2693	12,289	9045	620,641	61	865	34,038	44,148	0.21	192,423
Single testing for NG														
Status quo	40,639	46,877	227,626	315,142	–	9208	5806	620,467	–	–	54,279	–	–	192,296
12mNG^e^	41,181	36,401	229,895	307,477	−7665	14,071	5987	620,362	−105	1577	51,356	73,000	0.22	192,339
6mNG	40,835	41,118	228,449	310,402	2925	10,932	5899	620,429	67	2750	52,622	43,657	0.14	192,331
3mNG	40,568	41,132	227,337	318,297	7895	8590	5759	620,480	51	5106	55,265	154,804	−0.05	192,268
Single testing for Syphilis														
Status quo	51,336	4591	270,434	326,361	–	157,664	125,571	617,500	–	–	2599	–	–	191,249
12mSyphilis	66,951	3925	298,099	368,975	42,614	206,828	157,044	614,936	−2564	−867	2350	dominated	/	190,015
6mSyphilis	48,647	4726	264,665	318,038	−50,937	144,172	115,140	617,886	2950	−617	2762	cost-saving	42.73	191,454
3mSyphilis^e^	39,143	5993	240,260	285,396	−32,642	78,985	59,951	619,118	1232	−521	4760	cost-saving	21.52	192,168
Combined dual CT&NG + Syphilis testing														
Status quo	51,336	51,468	725,335	828,139	–	181,431	141,623	1,858,597	–	–	5884	–	–	577,177
12m (CT + NG) + 12mSyphilis	66,951	40,326	765,736	873,013	44,874	287,050	194,106	1,855,722	−2875	−425	4498	dominated	/	575,822
6m (CT + NG) + 12mSyphilis	66,951	45,044	756,491	868,485	−4528	243,801	178,936	1,855,946	224	−647	4854	cost-saving	4.85	575,938
3m (CT + NG) + 12mSyphilis	66,951	45,057	752,118	864,126	−4359	227,707	171,849	1,856,058	112	−778	5028	cost-saving	4.18	576,017
12m (CT + NG) + 6mSyphilis	48,647	41,127	732,302	822,076	−42,050	224,394	152,202	1,858,672	2614	142	5401	cost-saving	0.67	577,261
6m (CT + NG) + 6mSyphilis	48,647	45,844	723,057	817,548	−4528	181,145	137,032	1,858,896	224	−37032	5966	cost-saving	−0.41	577,377
3m (CT + NG) + 6mSyphilis	48,647	45,858	718,684	813,189	−4359	165,051	129,944	1,859,008	112	−913	6258	cost-saving	−1.19	577,456
12m (CT + NG) + 3mSyphilis	46,860	42,394	707,897	797,151	−16,038	159,207	97,013	1,859,904	896	−1395	8217	cost-saving	−1.92	577,898
6m (CT + NG) + 3mSyphilis	42,450	47,111	698,652	788,213	−8938	115,958	81,843	1,860,128	224	−610	9631	cost-saving	−6.12	578,058
3m (CT + NG) + 3mSyphilis^e^	40,929	47,125	694,279	782,332	−5881	99,864	74,756	1,860,239	111	−562	10,465	cost-saving	−7.15	578,152

CT = *Chlamydia trachomatis*. NG = *Neisseria gonorrhoeae*. ICER = incremental cost-effectiveness ratio. MSM = men who have sex with men. PrEP = pre-exposure prophylaxis. STI = sexually transmitted infections. TGW = transgender women. QALY = quality-adjusted life-year. WTP = willingness-to-pay.

Status quo of STI testing frequency: 3.22 times for CT/NG and 1.8 times for syphilis per year for Australian MSM, 0.2 times for CT/NG and 0.7 times for syphilis per year for Brazilian MSM/TGW, and 0.4 times for CT/NG/syphilis for Thailand MSM/TGW.

a Compared with the strategy producing the highest number of STI cases.

b Compared with 12-monthly STI testing strategy.

c BCR (benefit to cost ratio). In a certain STI testing strategy, BCR = number of dollars saved from STI treatment/number of dollars invested in STI testing.

d NMB (net monetary benefit) = effectiveness∗willingness to pay—cost.

e Preferred strategy under willingness-to-pay threshold.

**Fig. 1:**
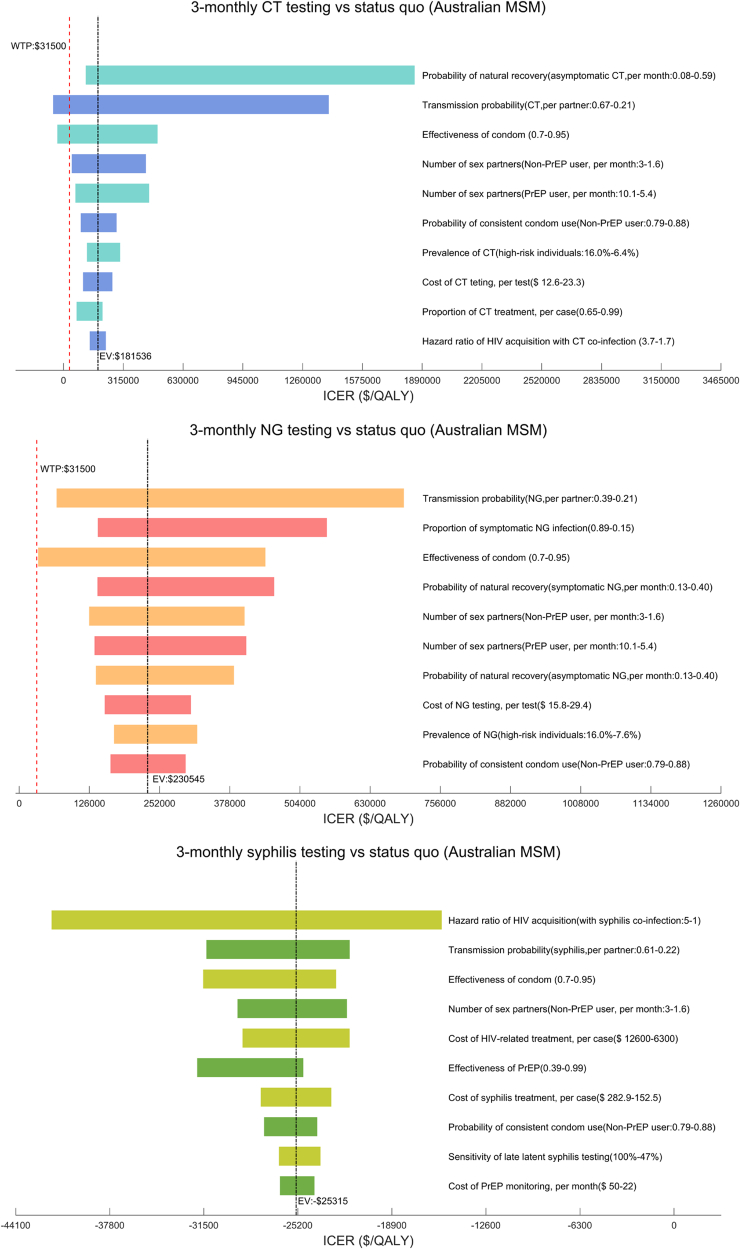
One-way sensivity analyses among Australian MSM.

One-way sensitivity analyses indicated the robustness of the cost-effectiveness of 3-monthly syphilis testing compared with the status quo. Testing for CT or NG every 3 months was also cost-effective than the status quo, with higher transmission probability of CT and lower effectiveness of condom use among MSM (Fig. 1). However, PSA suggested that under the current WTP, 3-monthly CT and annual NG testing have a higher probability of being cost-effective than the status quo (Figure S5, CT = 0.29 vs. 0.22, NG = 0.67 vs. 0.09).

### Brazil (willingness-to-pay threshold = $8786)

#### Men who have sex with men

For the single pathogen testing scenario for PrEP users, the optimal testing frequencies were every 6 months for CT (cost-saving, BCR = 2.95), maintaining the status quo (testing 0.2 times per year) for NG, and every 3 months for syphilis (cost-saving, BCR = 112.50). These strategies would identify 97,070 new CT cases, 10,829 new NG cases, and 542,242 new syphilis cases, with costs of $18,691, $163,651, and $3553 for per CT, NG, and syphilis case diagnosed, respectively. For the combined testing scenario, 6-monthly dual testing for CT/NG, along with 3-monthly separate syphilis testing (cost-saving, BCR = 10.82) was the optimal testing strategy. This strategy would identify 665,709 new STI cases with a cost of $8119 per STI case diagnosed (Table 2).

**Table 2:** Health, economic outcomes and cost-effectiveness of STI testing frequencies for PrEP users among Brazilian MSM (for 5 years).

Strategy	Cost, $, thousand					Effectiveness				Cost-effectiveness				
	PrEP related	STI testing	STI treatment	Total	Dif. cost	No. STI cases	No. identified STI cases	QALYs	Dif. QALYs	Cost per case averted, $^a^	Cost per case identified, $	ICER ($/QALY gained)^b^	BCR^c^	NMB^d^, per capita
Single testing for CT														
Status quo	57,463	6524	1,796,269	1,860,256	–	998,108	138,740	7,718,300	–	–	13,408	–	–	659,527
12mCT	51,456	15,181	1,752,851	1,819,488	−40,768	579,899	127,667	7,720,200	1900	−98	14,252	cost-saving	5.02	660,102
6mCT^e^	49,713	26,094	1,738,485	1,814,292	−5196	455,513	97,070	7,720,981	781	−85	18,691	cost-saving	2.95	660,222
3mCT	48,995	47,727	1,732,329	1,829,051	14,759	411,963	83,731	7,721,391	410	−54	21,844	35,998	1.55	660,111
Single testing for NG														
Status quo^e^	49,731	6454	1,715,979	1,772,164	–	105,379	10,829	7,721,009	–	–	163,651	–	–	660,646
12mNG	49,532	15,298	1,712,672	1,777,502	5338	92,998	20,148	7,721,227	218	432	88,220	24,486	0.37	660,612
6mNG	49,390	26,485	1,710,388	1,786,263	8761	84,774	26,397	7,721,387	160	685	67,668	54,756	0.28	660,538
3mNG	49,246	48,598	1,708,183	1,806,027	19,764	76,970	32,542	7,721,549	162	1192	55,498	122,000	0.18	660,355
Single testing for Syphilis														
Status quo	76,200	4449	2,042,794	2,123,443	–	1,943,418	1,046,348	7,624,837	–	–	2029	–	–	648,684
12mSyphilis	66,722	2509	1,996,050	2,065,281	−58,162	1,809,374	1,026,515	7,642,286	17,449	−434	2012	cost-saving	−24.09	650,798
6mSyphilis	50,853	3663	1,904,967	1,959,483	−105,798	1,294,162	714,189	7,668,450	26,164	−253	2744	cost-saving	−175.35	654,155
3mSyphilis^e^	46,634	5950	1,873,928	1,926,512	−32,971	1,081,967	542,242	7,674,789	6339	−229	3553	cost-saving	112.50	655,042
Combined dual CT&NG + Syphilis testing														
Status quo	76,200	10,973	5,555,043	5,642,216	–	3,046,905	1,195,917	23,064,146	–	–	4755	–	–	1,969,994
12m (CT + NG) + 12mSyphilis	66,722	17,807	5,461,572	5,546,101	−96,115	2,482,271	1,174,330	23,083,713	19,567	−171	4723	cost-saving	13.68	1,972,674
6m (CT + NG) + 12mSyphilis	66,722	28,994	5,444,923	5,540,639	−5462	2,483,705	1,149,982	23,084,653	940	−181	4818	cost-saving	6.11	1,972,811
3m (CT + NG) + 12mSyphilis	66,722	51,108	5,436,562	5,554,392	13,753	2,298,307	1,142,788	23,085,226	573	−118	4860	cost-saving	2.95	1,972,724
12m (CT + NG) + 6mSyphilis	51,456	18,961	5,370,489	5,440,907	−113,485	1,967,060	862,005	23,109,878	24,652	−187	6312	cost-saving	23.10	1,976,025
6m (CT + NG) + 6mSyphilis	50,853	30,148	5,353,842	5,434,842	−6065	1,834,449	837,657	23,110,818	940	−172	6488	cost-saving	10.49	1,976,168
3m (CT + NG) + 6mSyphilis	50,853	52,262	5,345,479	5,448,594	13,752	1,783,095	830,463	23,111,391	573	−154	6561	cost-saving	5.08	1,976,081
12m (CT + NG) + 3mSyphilis	51,456	21,248	5,339,450	5,412,154	−36,440	1,754,864	690,057	23,116,217	4826	−179	7843	cost-saving	20.98	1,976,869
6m (CT + NG) + 3mSyphilis^e^	49,713	32,435	5,322,802	5,404,949	−7205	1,622,254	665,709	23,117,157	940	−167	8119	cost-saving	10.82	1,977,024
3m (CT + NG) + 3mSyphilis	49,246	54,549	5,314,440	5,418,235	13,286	1,570,899	658,515	23,117,730	573	−152	8228	23,187	5.52	1,976,941

CT = *Chlamydia trachomatis*. NG = *Neisseria gonorrhoeae*. ICER = incremental cost-effectiveness ratio. MSM = men who have sex with men. PrEP = pre-exposure prophylaxis. STI = sexually transmitted infections. TGW = transgender women. QALY = quality-adjusted life-year. WTP = willingness-to-pay.

Status quo of STI testing frequency: 3.22 times for CT/NG and 1.8 times for syphilis per year for Australian MSM, 0.2 times for CT/NG and 0.7 times for syphilis per year for Brazilian MSM/TGW, and 0.4 times for CT/NG/syphilis for Thailand MSM/TGW.

a Compared with the strategy producing the highest number of STI cases.

b Compared with 12-monthly STI testing strategy.

c BCR (benefit to cost ratio). In a certain STI testing strategy, BCR = number of dollars saved from STI treatment/number of dollars invested in STI testing.

d NMB (net monetary benefit) = effectiveness∗willingness to pay—cost.

e Preferred strategy under willingness-to-pay threshold.

**Fig. 2:**
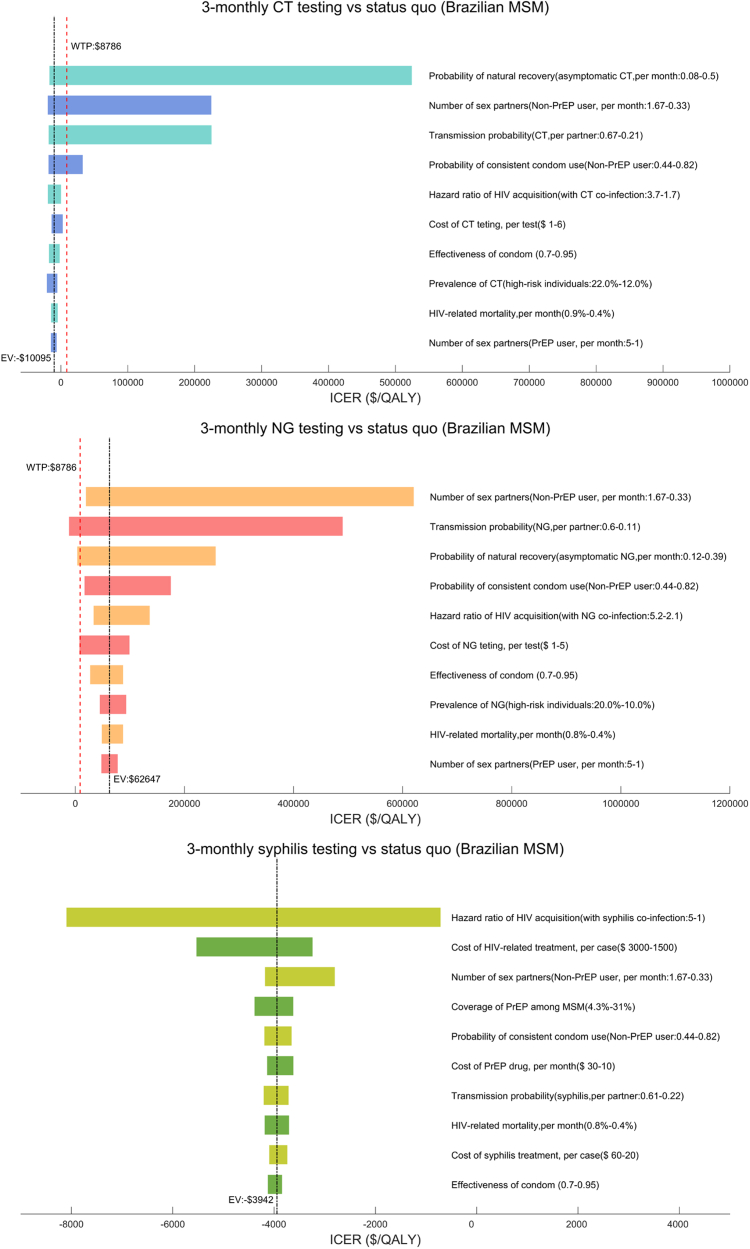
One-way sensivity analyses among Brazilian MSM.

One-way sensitivity analyses indicated the robustness of the cost-effectiveness of 3-monthly syphilis testing compared with the status quo. However, the cost-effectiveness of testing for CT or NG every 3 months was sensitive to several parameters, including the probability of natural CT/NG recovery, the number of sex partners of non-PrEP users, and the transmission probability of CT/NG (Fig. 2). PSA also suggested that under the current WTP ($8786), maintaining the status quo for CT/NG testing was more cost-effective than other strategies (Figure S6).

#### Transgender women

For the single pathogen testing scenario for PrEP users, the optimal frequencies were all every 3 months for CT (cost-saving, BCR = 3.27), NG (cost-saving, BCR = 1.79), and syphilis (cost-saving, BCR = 31.73). These strategies would identify 141,315 new CT cases, 94,300 new NG cases, and 195,901 new syphilis cases with costs of $1396, $2092, and $1132 per CT, NG, and syphilis cases diagnosed. For the combined testing scenario for CT, NG, and syphilis, the optimal testing frequency was 3-monthly dual testing for CT/NG, together with 3-monthly separate syphilis testing (cost-saving, BCR = 7.51). This strategy would identify 431,516 new STI cases for $1390 per STI case diagnosed (Table 3).

**Table 3:** Health, economic outcomes and cost-effectiveness of STI testing frequencies for PrEP users among Brazilian TGW (for 5 years).

Strategy	Cost, $, thousand					Effectiveness				Cost-effectiveness				
	PrEP related	STI testing	STI treatment	Total	Dif. cost	No. STI cases	No. identified STI cases	QALYs	Dif. QALYs	Cost per case averted, $^a^	Cost per case identified, $	ICER ($/QALY gained)^b^	BCR^c^	NMB^d^, per capita
Single testing for CT														
Status quo	7462	665	199,802	207,929	–	227,388	34,894	770,649		–	5959	–	–	65,630
12mCT	6925	1561	194,753	203,239	−4690	233,239	75,889	770,889	240	802	2678	cost-saving	5.64	65,698
6mCT	6527	2655	190,774	199,956	−3283	237,716	107,297	771,085	196	772	1864	cost-saving	4.54	65,748
3mCT^e^	6113	4765	186,409	197,287	−2669	242,503	141,315	771,306	221	705	1396	cost-saving	3.27	65,794
Single testing for NG														
Status quo	6056	705	194,152	200,913	–	198,186	19,200	770,488		–	10,464	–	–	65,686
12mNG	5931	1587	191,797	199,315	−1598	199,486	42,774	770,574	86	1230	4660	cost-saving	2.67	65,709
6mNG	5819	2691	189,596	198,106	−1209	200,688	64,877	770,657	83	1122	3054	cost-saving	2.29	65,729
3mNG^e^	5675	4846	186,720	197,241	−865	202,241	94,300	770,768	111	906	2092	cost-saving	1.79	65,747
Single testing for Syphilis														
Status quo	10,748	212	227,009	237,969	–	211,107	127,772	756,557		–	1862	–	–	64,091
12mSyphilis	10,055	254	224,247	234,556	−3413	217,940	143,331	758,196	1639	500	1636	cost-saving	65.76	64,270
6mSyphilis	8696	388	218,433	227,517	−7039	231,501	173,273	760,999	2803	513	1313	cost-saving	48.73	64,586
3mSyphilis^e^	7666	636	213,555	221,857	−5660	242,073	195,901	762,729	1730	521	1132	cost-saving	31.73	64,795
Combined dual CT&NG + Syphilis testing														
Status quo	10,748	917	620,964	632,629	–	636,681	181,866	2,297,694		–	3505	–	–	195,549
12m (CT + NG) + 12mSyphilis	10,055	1842	610,796	622,693	−9936	650,665	261,994	2,299,659	1965	711	2377	cost-saving	10.99	195,821
6m (CT + NG) + 12mSyphilis	10,055	2945	604,616	617,616	−5077	656,344	315,505	2,299,938	279	764	1958	cost-saving	8.06	195,896
3m (CT + NG) + 12mSyphilis	10,055	5100	597,376	612,531	−5085	662,685	378,946	2,300,270	332	773	1616	cost-saving	5.64	195,976
12m (CT + NG) + 6mSyphilis	8696	1975	604,983	615,654	3123	664,226	291,936	2,302,462	2192	617	2109	1425	15.10	196,138
6m (CT + NG)+ 6mSyphilis	8696	3078	598,802	610,577	−5077	669,905	345,447	2,302,741	279	664	1767	cost-saving	10.26	196,213
3m (CT + NG) + 6mSyphilis	8696	5234	591,562	605,492	−5085	676,246	408,888	2,303,073	332	686	1481	cost-saving	6.81	196,293
12m (CT + NG) + 3mSyphilis	7666	2223	600,105	609,994	4502	674,798	314,564	2,304,192	1119	594	1939	4023	15.97	196,346
6m (CT + NG) + 3mSyphilis	7666	3326	593,925	604,917	−5077	680,477	368,075	2,304,471	279	633	1643	cost-saving	11.22	196,422
3m (CT + NG) + 3mSyphilis^e^	7666	5482	586,684	599,832	−5085	686,818	431,516	2,304,803	332	655	1390	cost-saving	7.51	196,502

CT = *Chlamydia trachomatis*. NG = *Neisseria gonorrhoeae*. ICER = incremental cost-effectiveness ratio. MSM = men who have sex with men. PrEP = pre-exposure prophylaxis. STI = sexually transmitted infections. TGW = transgender women. QALY = quality-adjusted life-year. WTP = willingness-to-pay.

Status quo of STI testing frequency: 3.22 times for CT/NG and 1.8 times for syphilis per year for Australian MSM, 0.2 times for CT/NG and 0.7 times for syphilis per year for Brazilian MSM/TGW, and 0.4 times for CT/NG/syphilis for Thailand MSM/TGW.

a Compared with the strategy producing the highest number of STI cases.

b Compared with 12-monthly STI testing strategy.

c BCR (benefit to cost ratio). In a certain STI testing strategy, BCR = number of dollars saved from STI treatment/number of dollars invested in STI testing.

d NMB (net monetary benefit) = effectiveness∗willingness to pay—cost.

e Preferred strategy under willingness-to-pay threshold.

**Fig. 3:**
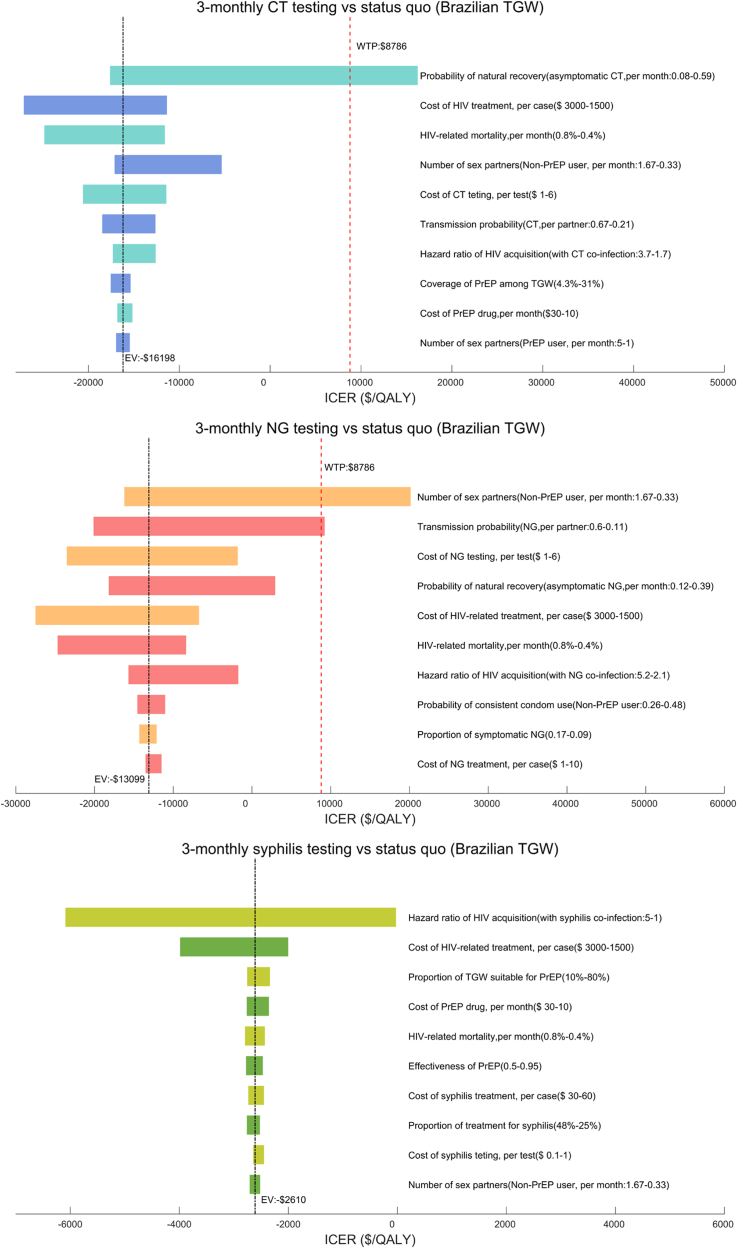
One-way sensivity analyses among Brazilian TGW.

One-way sensitivity analyses demonstrated the robustness of the cost-effectiveness of 3-monthly syphilis testing compared with the status quo. The cost-effectiveness of 3-monthly CT testing was sensitive to the probability of natural CT recovery, while 3-monthly NG testing was sensitive to the number of sex partners of non-PrEP users (Fig. 3). PSA suggested that under the current WTP ($8786), 3-monthly CT/NG/syphilis testing still had a higher probability of being cost-effective than other strategies (Figure S7).

### Thailand (willingness-to-pay threshold = $4800)

#### Men who have sex with men

For the single pathogen testing scenario for PrEP users, the optimal testing frequencies were maintaining the status quo (testing 0.4 times per year) for both CT and NG, and every 6 months for syphilis (ICER = $1579/QALY gained, BCR = 0.54). These strategies would identify 22,430 new CT cases, 4002 new NG cases, and 69,750 new syphilis cases, with costs of $5905, $32,416, and $1730 per CT, NG, and syphilis case diagnosed, respectively. For the combined testing scenario, the optimal testing frequency was annual dual testing for CT/NG, along with 6-monthly syphilis testing (cost-saving, BCR = 0.27). This strategy would identify 97,275 new STI cases and cost $3166 for each STI case diagnosed (Table 4).

**Table 4:** Health, economic outcomes and cost-effectiveness of STI testing frequencies for PrEP users among Thailand MSM (for 5 years).

Strategy	Cost, $, thousand					Effectiveness				Cost-effectiveness				
	PrEP related	STI testing	STI treatment	Total	Dif. cost	No. STI cases	No. identified STI cases	QALYs	Dif. QALYs	Cost per case averted, $^a^	Cost per case identified, $	ICER ($/QALY gained)^b^	BCR^c^	NMB^d^, per capita
Single testing for CT														
Status quo^e^	28,129	33,662	70,665	132,456	–	86,385	22,430	3,139,628	–	–	5905	–	–	149,378
12mCT	27,444	46,166	70,042	143,652	11,196	68,291	22,122	3,139,742	114	619	6494	98,211	0.05	149,271
6mCT	27,040	67,135	69,630	163,805	20,153	58,286	21,305	3,139,828	86	1116	7689	234,337	0.03	149,074
3mCT	26,791	108,565	69,374	204,730	40,925	52,600	20,740	3,139,889	61	2140	9871	670,902	0.02	148,667
Single testing for NG														
Status quo^e^	26,684	33,545	69,490	129,719	–	23,569	4002	3,139,791	–	–	32,416	–	–	149,413
12mNG	26,635	46,110	69,412	142,157	12,438	22,398	5403	3,139,812	21	10,622	26,309	592,286	0.01	149,289
6mNG	26,581	67,181	69,329	163,091	20,934	21,160	6871	3,139,835	23	13,854	23,735	910,174	0.00	149,081
3mNG	26,522	108,834	69,241	204,597	41,506	19,873	8374	3,139,860	25	20,260	24,431	1,660,240	0.00	148,667
Single testing for Syphilis														
Status quo	32,417	11,276	79,042	122,735	–	326,611	123,029	3,123,944	–	–	998	–	–	148,722
12mSyphilis	27,574	15,092	75,536	118,202	−4533	246,672	93,852	3,128,450	4506	−57	1259	cost-saving	0.92	148,984
6mSyphilis^e^	25,733	21,350	73,601	120,684	2482	208,079	69,750	3,130,022	1572	−18	1730	1579	0.54	149,034
3mSyphilis	25,319	33,983	73,051	132,353	11,669	199,969	63,257	3,130,351	329	76	2092	35,468	0.26	148,933
Combined dual CT&NG + Syphilis testing														
Status quo	32,417	44,938	219,197	296,552	–	436,564	83,997	9,403,363	–	–	3531	–	–	448,396
12m (CT + NG)+12mSyphilis	27,574	61,258	214,990	303,822	7270	337,362	121,377	9,408,004	4641	74	2503	1566	0.26	448,546
6m (CT + NG)+12mSyphilis	27,574	82,273	214,495	324,342	20,520	326,119	122,028	9,408,114	110	252	2658	186,545	0.13	448,346
3m (CT + NG)+12mSyphilis	27,574	123,926	214,151	365,651	41,309	319,145	122,967	9,408,199	85	589	2974	485,988	0.06	447,937
12m (CT + NG)+6mSyphilis^e^	27,444	67,516	213,055	308,015	−57,636	298,769	97,275	9,409,576	1377	84	3166	cost-saving	0.27	448,579
6m (CT + NG)+6mSyphilis	27,040	88,531	212,560	328,131	20,116	287,526	97,926	9,409,685	109	212	3351	184,550	0.15	448,384
3m (CT + NG)+6mSyphilis	26,791	130,184	212,216	369,191	41,060	280,551	98,864	9,409,771	86	466	3734	477,442	0.08	447,977
12m (CT + NG)+3mSyphilis	27,444	80,149	212,504	320,097	−49,094	290,659	90,782	9,409,905	134	162	3526	cost-saving	0.19	448,474
6m (CT + NG)+3mSyphilis	27,040	101,164	212,010	340,214	20,117	279,416	91,433	9,410,014	109	278	3721	184,560	0.13	448,279
3m (CT + NG)+3mSyphilis	26,791	142,817	211,666	381,274	41,060	272,442	92,372	9,410,100	86	517	4128	477,442	0.08	447,872

CT = *Chlamydia trachomatis*. NG = *Neisseria gonorrhoeae*. ICER = incremental cost-effectiveness ratio. MSM = men who have sex with men. PrEP = pre-exposure prophylaxis. STI = sexually transmitted infections. TGW = transgender women. QALY = quality-adjusted life-year. WTP = willingness-to-pay.

Status quo of STI testing frequency: 3.22 times for CT/NG and 1.8 times for syphilis per year for Australian MSM, 0.2 times for CT/NG and 0.7 times for syphilis per year for Brazilian MSM/TGW, and 0.4 times for CT/NG/syphilis for Thailand MSM/TGW.

a Compared with the strategy producing the highest number of STI cases.

b Compared with 12-monthly STI testing strategy.

c BCR (benefit to cost ratio). In a certain STI testing strategy, BCR = number of dollars saved from STI treatment/number of dollars invested in STI testing.

d NMB (net monetary benefit) = effectiveness∗willingness to pay—cost.

e Preferred strategy under willingness-to-pay threshold.

**Fig. 4:**
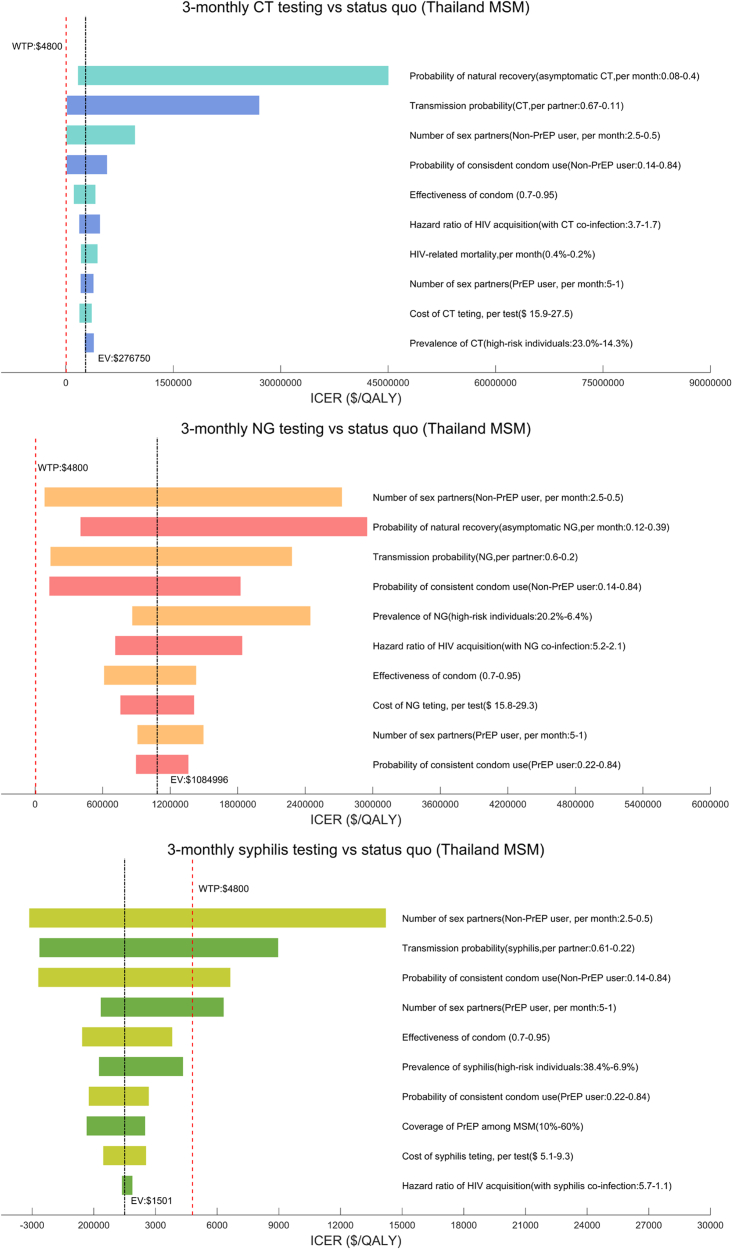
One-way sensivity analyses among Thailand MSM.

One-way sensitivity analyses indicated the robustness of the cost-effectiveness of maintaining the status quo for CT or NG testing compared with the 3-monthly testing strategy. Testing for syphilis every 3 months was less cost-effective with lower transmission probability, fewer sex partners, and lower probability of condom use among non-PrEP users (Fig. 4). PSA suggested that under the current WTP ($4800), maintaining the status quo for CT and NG still had a higher probability of being cost-effective than other strategies, while 3-monthly syphilis testing has a lower probability of being cost-effective than 6-monthly testing (0.26 vs. 0.38) (Figure S8).

#### Transgender women

For the single pathogen testing scenario, the optimal testing frequencies were maintaining the status quo (0.4 times testing per year) for both CT and NG, and every 3 months for syphilis (ICER = $3063/QALY gained, BCR = 0.16). These strategies would identify 25,405 new CT cases, 12,350 new NG cases, and 33,415 new syphilis cases, with costs of $626, $1152, and $464 per CT, NG, and syphilis case diagnosed, respectively. For the combined testing scenario, the optimal testing frequency was annual dual testing for CT/NG, along with 3-monthly syphilis testing (cost-saving, BCR = 0.18). This strategy would identify 88,443 new STI cases, with a cost of $400 per STI case diagnosed (Table 5).

**Table 5:** Health, economic outcomes and cost-effectiveness of STI testing frequencies for PrEP users among Thailand TGW (for 5 years).

Strategy	Cost, $, thousand					Effectiveness				Cost-effectiveness				
	PrEP related	STI testing	STI treatment	Total	Dif. cost	No. STI cases	No. identified STI cases	QALYs	Dif. QALYs	Cost per case averted, $^a^	Cost per case identified, $	ICER ($/QALY gained)^b^	BCR^c^	NMB^d^, per capita
Single testing for CT														
Status quo^e^	5470	2693	7742	15,905	–	94,158	25,405	231,959	–	–	626	–	–	10,975
2m_CT	5173	4112	7534	16,819	914	96,129	36,598	232,003	44	−464	460	20,773	0.15	10,968
6m_CT	4850	6407	7292	18,549	1730	98,314	49,046	232,054	51	−637	378	33,922	0.12	10,953
3m_CT	4508	10,790	7016	22,314	3765	100,692	62,723	232,114	60	−981	356	62,750	0.09	10,918
Single testing for NG														
Status quo^e^	4350	2677	7204	14,231	–	68,916	12,350	232,006	–	–	1152	.	–	10,994
12m_NG	4281	4076	7120	15,477	1246	69,269	18,430	232,027	21	−3530	840	59,333	0.06	10,983
6m_NG	4193	6391	7011	17,595	2118	69,722	26,234	232,055	28	−4174	671	75,643	0.05	10,963
3m_NG	4081	10,903	6863	21,847	4252	70,314	36,410	232,094	39	−5448	600	109,026	0.04	10,922
Single testing for Syphilis														
Status quo	5422	937	8313	14,672	–	38,697	20,906	229,275	–	–	702	–	–	10,858
12m_Syphilis	4827	1433	8174	14,434	−238	40,306	26,255	229,991	716	148	550	cost-saving	0.28	10,895
6m_Syphilis	4358	2187	8025	14,570	136	41,606	30,360	230,471	480	36	480	283	0.23	10,917
3m_Syphilis^e^	4012	3596	7884	15,492	922	42,605	33,415	230,772	301	−210	464	3063	0.16	10,922
Combined dual CT&NG + Syphilis testing														
Status quo	5470	3630	23,260	32,360	–	201,771	58,661	693,240	–	–	552	–	–	32,952
12m_(CT + NG) + 12m_Syphilis	5173	5545	22,828	33,546	1186	205,704	81,284	694,021	781	−302	413	1519	0.23	32,978
6m_(CT + NG) + 12m_Syphilis	4850	7839	22,477	35,166	1620	208,342	101,536	694,101	80	−428	346	20,250	0.19	32,965
3m_(CT + NG) + 12m_Syphilis	4827	12,336	22,053	39,217	4051	211,312	125,389	694,199	98	−719	313	41,337	0.14	32,929
12m_(CT + NG) + 6m_Syphilis	5173	6299	22,679	34,151	−5066	207,004	85,388	694,501	302	−343	400	cost-saving	0.22	32,995
6m_(CT + NG) + 6m_Syphilis	4850	8593	22,328	35,771	1620	209,642	105,640	694,581	80	−434	339	20,250	0.19	32,982
3m_(CT + NG) + 6m_Syphilis	4508	13,090	21,904	39,502	3731	212,612	129,493	694,680	99	−659	305	37,687	0.14	32,950
12m_(CT + NG) + 3m_Syphilis^e^	5173	7708	22,538	35,420	−4082	208,003	88,443	694,801	121	−492	400	cost-saving	0.18	32,996
6m_(CT + NG) + 3m_Syphilis	4850	10,002	22,187	37,039	1619	210,641	108,696	694,881	80	−528	341	20,238	0.17	32,984
3m_(CT + NG) + 3m_Syphilis	4508	14,499	21,764	40,771	3732	213,611	132,549	694,980	99	−711	308	37,697	0.14	32,951

CT = *Chlamydia trachomatis*. NG = *Neisseria gonorrhoeae*. ICER = incremental cost-effectiveness ratio. MSM = men who have sex with men. PrEP = pre-exposure prophylaxis. STI = sexually transmitted infections. TGW = transgender women. QALY = quality-adjusted life-year. WTP = willingness-to-pay.

Status quo of STI testing frequency: 3.22 times for CT/NG and 1.8 times for syphilis per year for Australian MSM, 0.2 times for CT/NG and 0.7 times for syphilis per year for Brazilian MSM/TGW, and 0.4 times for CT/NG/syphilis for Thailand MSM/TGW.

a Compared with the strategy producing the highest number of STI cases.

b Compared with 12-monthly STI testing strategy.

c BCR (benefit to cost ratio). In a certain STI testing strategy, BCR = number of dollars saved from STI treatment/number of dollars invested in STI testing.

d NMB (net monetary benefit) = effectiveness∗willingness to pay—cost.

e Preferred strategy under willingness-to-pay threshold.

**Fig. 5:**
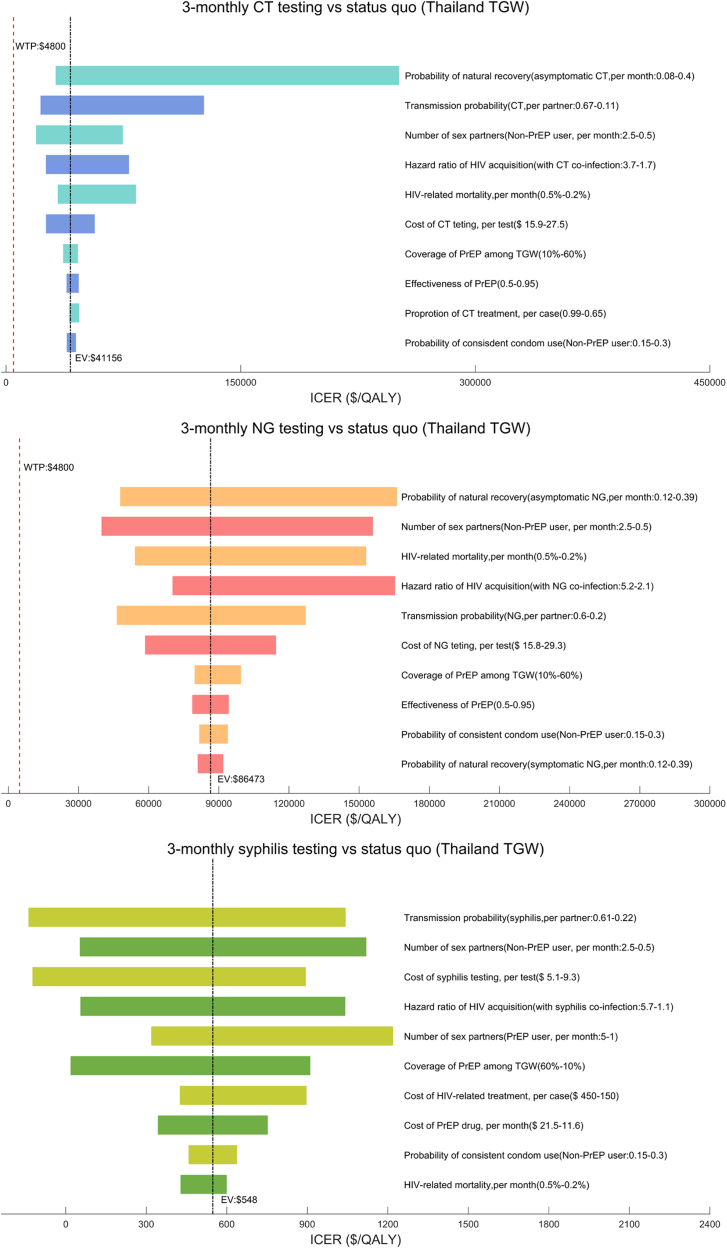
One-way sensivity analyses among Thailand TGW.

One-way sensitivity analyses demonstrated the robustness of the cost-effectiveness of maintaining the status quo for CT/NG testing compared with 3-monthly testing, and the cost-effectiveness of 3-monthly syphilis testing compared with the status quo (Fig. 5). PSA indicated that 6-monthly syphilis testing was more cost-effective when WTP<$2400, while 3-monthly syphilis testing became the most cost-effective strategy under the current WTP ($4800) (Figure S9).

## Discussion

The optimal frequency of STI testing should reflect context-specific factors, including underlying disease burden, behavioral patterns, health-system capacity, and associated costs. In this study, we developed models to evaluate the health impact and cost-effectiveness of alternative STI testing strategies for chlamydia, gonorrhea, and syphilis among MSM and TGW using PrEP in Australia, Brazil, and Thailand. The selection of these three countries was made on the basis of their PrEP-friendly policies in both developed and developing settings, where PrEP is either provided free of charge or with financial assistance, thereby representing different stages of PrEP implementation and STI testing services among MSM and TGW. Such infrastructure and a longstanding commitment to PrEP delivery create favorable conditions for examining integrated PrEP–STI testing strategies. The practical experience and available data from these settings also offer valuable real-world insights, strengthening the feasibility and broader applicability of combined testing approaches across diverse economic contexts. By identifying optimal single-pathogen or combined testing intervals for the three STIs among MSM and TGW in Australia, Brazil, and Thailand, our findings provide actionable evidence to support decision-makers in selecting STI testing strategies that align with local health-care resources.

Our study showed that although more frequent STI testing substantially increases testing-related expenditures, it can reduce downstream treatment costs among MSM in Australia, Brazil, and Thailand. Increased testing frequency also prevented additional STI cases in all three countries, indicating that earlier detection and treatment could interrupt onward transmission. However, our modeling also demonstrated that, in most scenarios, only more frequent (3-monthly) syphilis testing was cost-effective. In contrast, increasing CT or NG testing frequency considerably raised costs, resulting in less favorable QALY gains and making these strategies less affordable for PrEP users and healthcare systems.^8^ The higher financial burden associated with intensified STI testing remains a major constraint in implementing etiologic STI services in LMICs such as Thailand.^17^^,^^18^ Moreover, disparities in access to sexual health care persist even in high-income countries with publicly funded systems, such as Australia. MSM and TGW continue to face barriers to obtaining appropriate STI testing and treatment, alongside the limited availability of gender-affirming health services.^56^

For single-pathogen CT/NG testing strategies, our modeling indicated that maintaining the status quo or adopting less frequent testing (every 6 or 12 months) was more cost-effective over a 5-year time horizon under current WTP thresholds for Australian MSM, Brazilian MSM, and Thai MSM/TGW. In contrast, more frequent syphilis testing (every 3 months) was identified as the optimal strategy for Australian and Brazilian MSM. Because syphilis has a longer duration of untreated infection, more severe clinical consequences than CT and NG, and lower testing costs, increasing testing frequency facilitates earlier detection and treatment of new infections. However, more frequent syphilis testing may require shortening the intervals between PrEP follow-up visits, which could discourage uptake and adherence to both PrEP use and STI testing among PrEP users.^57^^,^^58^

For practical and cost considerations, we also assessed the cost-effectiveness of dual and combined CT/NG testing, as well as separate syphilis testing. The results showed that 3-monthly dual CT/NG testing combined with 3-monthly syphilis testing was cost-effective in Australia and Brazil, indicating that frequent combined testing for all three pathogens could offer substantial economic benefits. However, implementation feasibility is not guaranteed. A recent systematic review of PrEP users reported reluctance toward 3-monthly testing because follow-up visits were viewed as time-consuming and inconvenient.^59^ Developing more affordable CT/NG testing options and decentralized syphilis testing approaches for PrEP users is therefore critical. Innovative strategies such as pay-it-forward have increased CT/NG testing uptake by 76.7% while reducing per capita costs by a factor of 13.2 among female sex workers.^60^ Syphilis self-testing may also be a promising approach, especially when using a dual HIV and syphilis rapid test in a single device.^61^^,^^62^ Both separate and dual syphilis self-testing are highly accepted and used, particularly among populations with limited access to public health services.^63^^,^^64^ In addition, dried blood spot sampling has been shown to be a valid and acceptable alternative to venous blood collection for STI testing, suitable for routine clinic care and for self-collection at home.^65^

Our analysis also supported 3-monthly syphilis testing for TGW in Brazil and Thailand. Expanding STI testing services for TGW is essential because they experience a disproportionately high burden of STIs compared with MSM, with STI prevalence estimated to be 9–36 times higher among TGW.^66^^,^^67^ However, reaching TGW who are marginalized and have inadequate access to STI services remains challenging. Studies from Brazil show that TGW face high levels of violence, stigma, and discrimination, which substantially limit their access to and retention in health and social services.^68^^,^^69^ Further research has highlighted insufficient transgender health knowledge among providers and a persistent gap between trans inclusive policies and their implementation in practice, further restricting access to care.^70^ Unlike MSM, for whom less frequent testing could avert more STI cases, we found that 3-monthly combined CT, NG, and syphilis testing produced the highest number of detected STI cases among TGW in Brazil and Thailand. In our model, we included only TGW who have sex with cisgender men, meaning that their STI risk was dependent on STI prevalence among cisgender men. Under this assumption, our findings suggest that increasing STI testing frequency alone among TGW, without simultaneously addressing the source of transmission from MSM and men who have sex with women, would not substantially reduce new STI infections.

Our study has several limitations. First, to simplify the model, we did not account for co-infections with two or more of the three STI pathogens (CT, NG, and syphilis), which likely produced conservative estimates of infections averted and associated costs because the model did not capture the possibility of detecting and treating multiple infections in the same individual. In addition, the model did not incorporate antimicrobial resistance to current first-line treatments. Second, we assumed stable sexual mixing between populations, which may have under- or over-estimated true STI transmission among MSM and TGW. Third, we applied overall utility values for being infected with an STI rather than utilities specific to individual complications such as prostatitis from CT or NG, or neurosyphilis. This may have underestimated the health impact of these infections. Fourth, although we simulated multiple testing patterns and frequencies, we did not incorporate other emerging interventions such as doxycycline post-exposure prophylaxis (doxyPEP) for bacterial STI prevention. Doxycycline, which is effective against CT, syphilis, and in some regions NG, can be taken after a sexual exposure that carries STI risk. Similar to PrEP for HIV prevention, several trials have demonstrated strong efficacy of doxyPEP for preventing CT and syphilis and moderate efficacy for NG.^71^ This strategy could reduce the need for frequent STI testing in some settings. However, concerns remain that widespread doxyPEP use may contribute to rising NG antimicrobial resistance, making doxyPEP an effective but temporary solution for reducing NG infections and burden.^72^^,^^73^ With increasing concern about resistance to doxycycline and ceftriaxone, which are the primary treatments for CT and NG in Australia, Brazil, and Thailand, future modeling studies should incorporate resistance dynamics as a key factor.^74^^,^^75^ This would allow exploration of more realistic and context-specific STI prevention and treatment strategies, thereby strengthening public health decision-making. Finally, we modeled only three countries, which limits the generalizability of our findings beyond the intended purpose of demonstrating the feasibility of estimating optimal testing frequencies for diverse populations, pathogens, and health system contexts. We plan to make this model broadly accessible so that other countries can input their own data to identify optimal STI testing frequencies.

In conclusion, our dynamic transmission models highlight the need for population- and context-specific STI testing strategies among MSM and TGW using PrEP. Optimal testing frequencies varied across settings, shaped by differences in epidemiology, health system capacity, and cost-effectiveness. For MSM, maintaining current CT or NG testing levels or testing less often, together with more frequent syphilis testing, provided the best balance of cost and health benefit. For TGW, distinct optimal patterns underscore the need for targeted approaches that address their unique vulnerabilities. Overall, flexible and contextually tailored STI testing strategies are essential to maximize health outcomes and use resources efficiently.

## Contributors

Rui Zhao, Maeve Brito de Mello and Jason J. Ong were involved in study concept and design, data acquisition, data analysis, interpretation of data, and drafting of the manuscript. Lei Zhang and Jason J. Ong were involved in the acquisition of data and interpretation of data. Pâmela Cristina Gaspar, Angelica Espinosa Miranda and Nittaya Phanuphak were involved in the interpretation of data and critical revision of the manuscript. Philippe Mayaud, Angela Carvalho Freitas, Katia Cristina Bassichetto, Maria Amelia Veras, Daniel McCartney, Jiajun Sun, Filip Meheus and Hao Lai were involved in the critical revision of the manuscript. Lei Zhang and Jason J. Ong were involved in study concept and design, interpretation of data, critical revision of the manuscript, and overall study supervision. Rui Zhao, Maeve Brito de Mello, Jason J. Ong and Lei Zhang had access to and verified the data. All authors participated in preparing the manuscript and have seen and approved the final version for submission.

## Data sharing statement

All data relevant to the study are included in the Article or in the online appendix.

## Declaration of interests

Maeve Brito de Mello reports Bill and Melinda Gates Foundation grant to the World Health Organization (WHO). All other authors declare no competing interests. Some of the authors are staff members of WHO. The authors alone are responsible for the views expressed in this publication and they do not necessarily represent the views, decisions, or policies of the WHO.
