# Supplementary material for: Optimal testing frequency for sexually transmitted infections among men who have sex with men and transgender women who use HIV pre-exposure prophylaxis in Australia, Brazil and Thailand: a cost-effectiveness analysis
Source: Lancet Reg Health West Pac. 2026 Apr 1;69:101837. doi: 10.1016/j.lanwpc.2026.101837 (PMC13084425; doi:10.1016/j.lanwpc.2026.101837)
Supplement: Supplementary Materials [file mmc1.docx]

**Supplementary materials**

***Transmission process of Chlamydia trachomatis (CT), Neisseria gonorrhoeae (NG) and syphilis***

Current studies usually assumed that exposed and infected persons have the same infectiousness, without explicitly distinguishing and separately parameterizing the infectiousness of latent and non-latent periods^1,2^. Given this limitation, we also assumed the infectivity of STIs identical during the latent and non-latent stages, and constructed dynamic transmission models based on a susceptible-infectious-recovery-susceptible (SIRS) model structure among MSM/TGW. CT, NG and syphilis models were included for individuals susceptible for HIV infection. We used separate models of STIs (CT, NG and syphilis) to simulate the transmission and progression of disease once an individual is infected with STI.

The transmission of these three STIs is mainly determined by force-of-infection (λ), which is the key parameter that takes into account the transmission probability, population prevalence, high-risk behavioural pattern and frequency of testing for the specific STI. To simplify our model, we did not take co-infections among these three STIs into consideration. At each monthly cycle length (time step), the transmission probability of CT/NG/syphilis per partner (β), the dynamic CT/NG/syphilis prevalence among MSM/TGW, the number of sex partners per individual has per year, and the coverage and efficacy of condom use against STI per anal sex were applied to infer λ per partnership for CT/NG/syphilis transmission. For MSM, the prevalence of STI derived from the STI prevalence among MSM, which would change in our dynamic model with the number of STI cases and the number of susceptible individuals. According to a survey in US in 2023, over 50% of TGW reported sex with cisgender men^3^. Therefore, we only modelled the STI epidemic for TGW who have sex with men in our study. The prevalence of STI among TGW derived from the STI prevalence among cisgender men in each country , which was assumed stable in the 5-year time horizon in our model. See the formula below for λ of CT/NG/syphilis:

$$\lambda=\beta\times{Prevalence}_{STI}\times{Number}_{sex partners}\times(1-{Condom}_{coverage}\times{Condom}_{efficacy})$$

The CT/NG models classified individuals as being susceptible (S; STI uninfected), infectious (I, STI infected) and recovered (R). Individuals who became infected with NG or CT were moved to either the asymptomatic or symptomatic infectious compartment. We assumed symptomatic and asymptomatic infections are equally infectious for CT/NG. Infected individuals would return to the recovered compartment due to either natural clearance of the pathogen or treatment. At any stage of CT/NG infection, if an individual was tested, they were assumed to be treated and returned to the susceptible compartment after recovery (Figure S1).


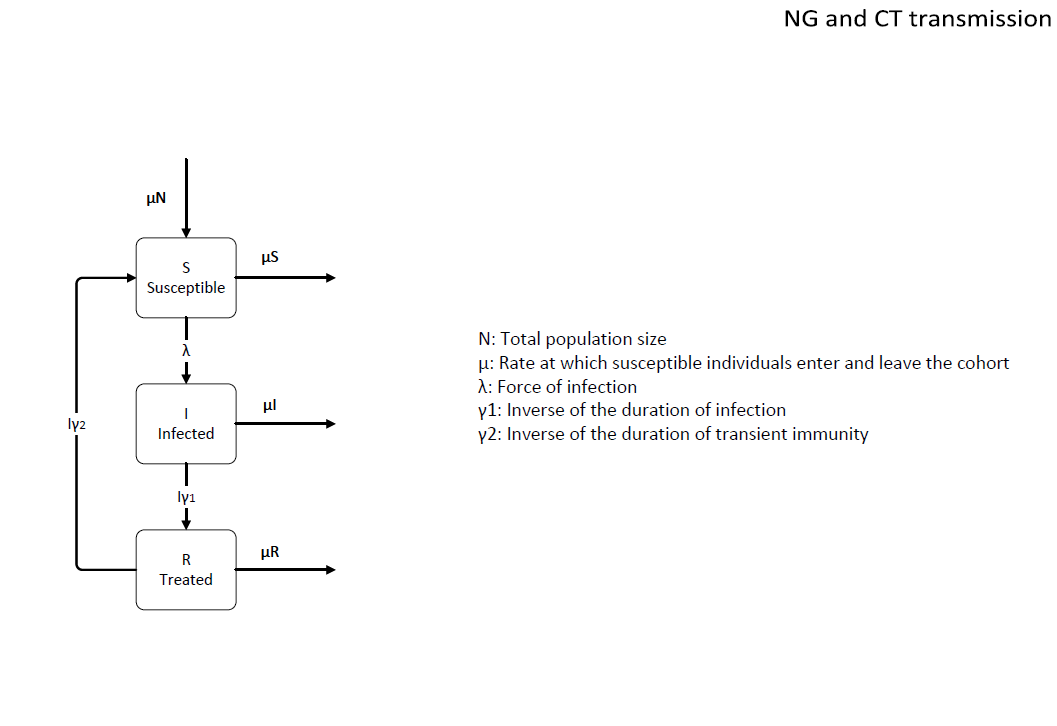


Figure S1 Transmission process of CT/NG among MSM/TGW

The syphilis model classified individuals as being susceptible (S; STI uninfected), infectious (Y1, in the primary syphilis disease state; Y2, in the secondary syphilis disease state; L1, in the early latent syphilis disease state; L2, in the late latent syphilis disease state, Y3, in the tertiary syphilis disease state) and recovered due to treatment (T). We assume that only individuals in the primary secondary, and early latent stages of syphilis infection are infectious. Individuals who became infected with syphilis, unless tested, were moved to the infectious compartment of early stages (comprising of primary, secondary and early latent syphilis), then progressed to the late stages of late latent and tertiary syphilis. At any stage of syphilis infection, if an individual was tested, they were assumed to be treated and returned to the susceptible compartment (Figure S2).


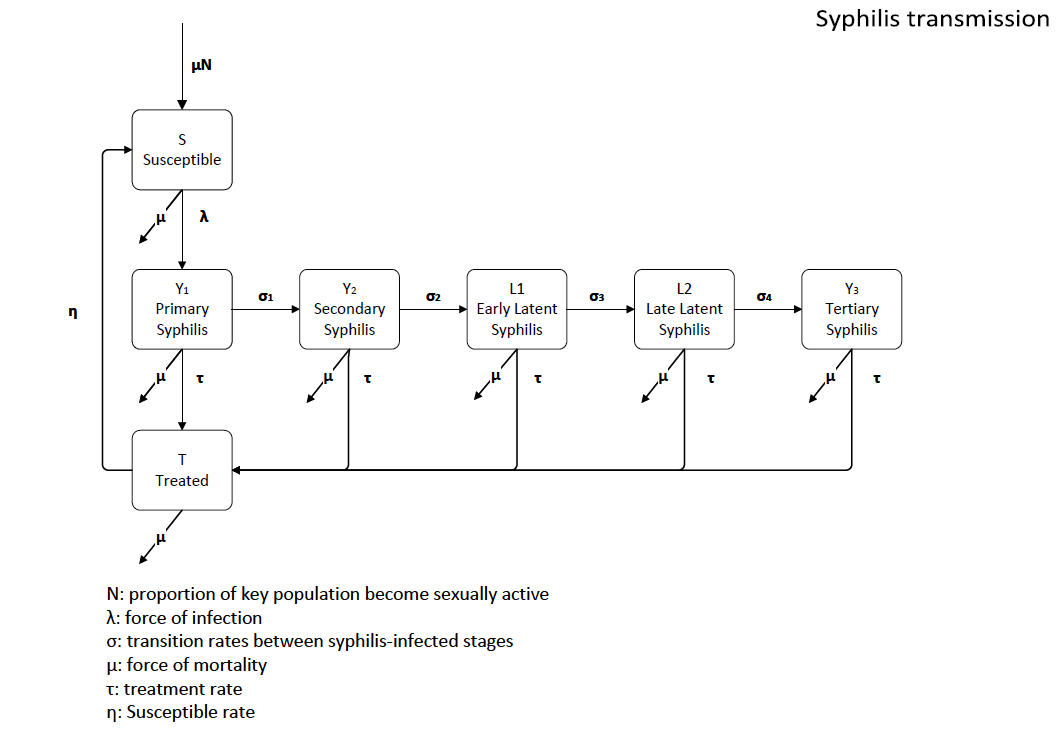


Figure S2 Transmission process of syphilis among MSM/TGW

***Markov process of CT, NG and syphilis***


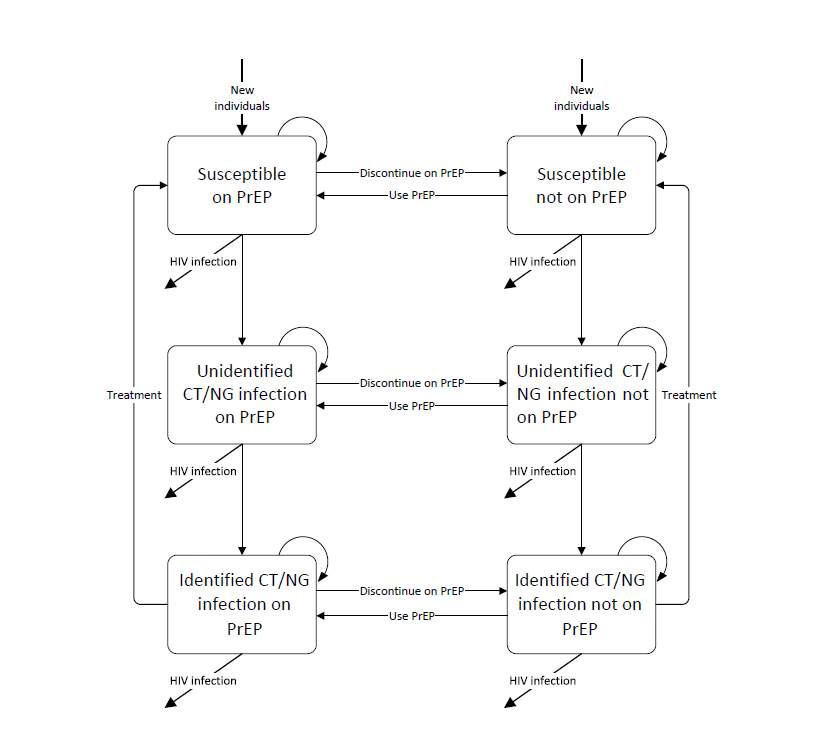


Figure S3 Markov process of CT/NG infection among MSM/TGW


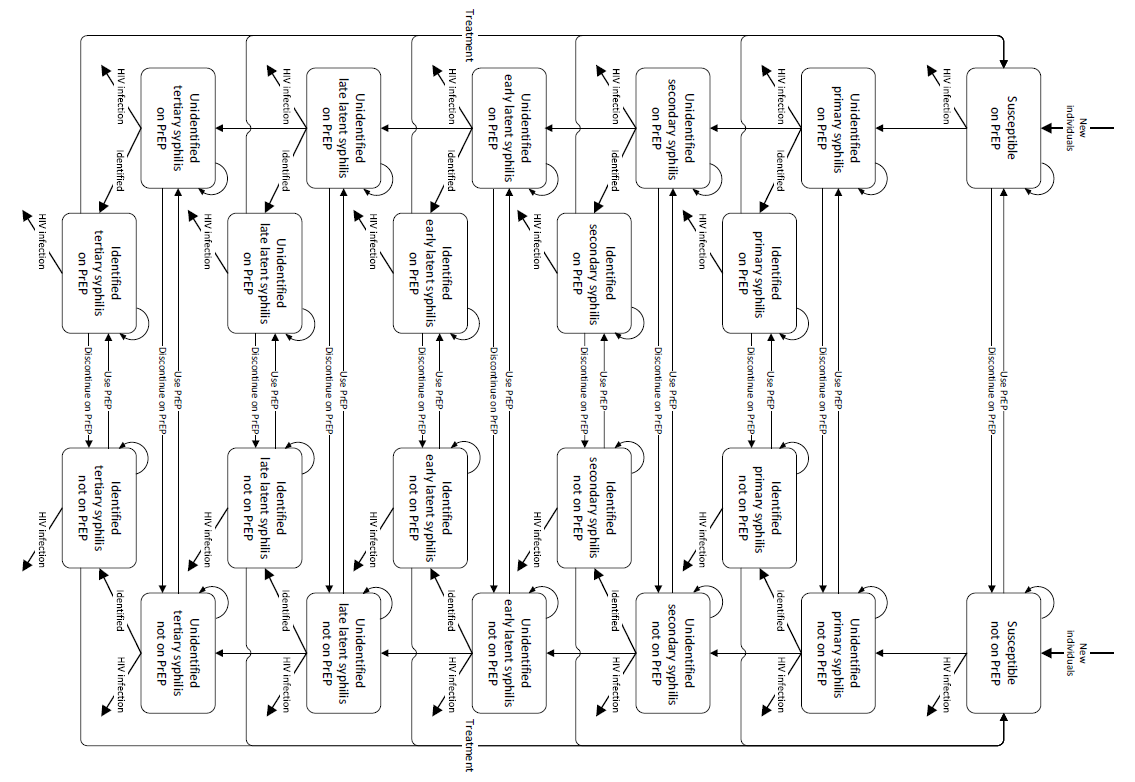


Figure S4 Markov process of syphilis infection among MSM/TGW

***Population size***

The actual population sizes of MSM/TGW, including the total population, those suitable for PrEP and those on PrEP in Australia, Thailand and Brazil are unclear. As the data of TGW was hard to obtain in Australia, we only estimated the population size of MSM. For Australian MSM, first we used the number of males between ages of 16-69 (8,292,223) and the proportion of males being MSM (1.88%) to obtain the number of MSM (155,894) based on a national study that estimated number of potential PrEP users among MSM in Australia^4^. Then according to the proportion of MSM suitable for PrEP (37.4%) and the uptake rate of PrEP (57%) based on the HIV, viral hepatitis and sexually transmissible infections in Australia annual surveillance report 2021 to obtain the number of MSM on PrEP (33,233) in Australia^5^. For Thailand MSM, we used the number of estimated national MSM (605,932) and TGW (138,433) as the total population, and the number of national MSM (123,004) and TGW (9,770) that meet the PrEP eligibility as the population suitable for PrEP based on the Estimation of PrEP targets for key and high-risk populations in Thailand, 2020-2022^6^. Then using the proportion of PrEP uptake among MSM (48%) and TGW (43.9%) based on the Thailand's largest KP-led PrEP programme “Princess PrEP” to obtain the number of PrEP users among MSM (59,042) and Thailand (4,289)^7^. For Brazilian MSM, we used the number of males between ages of 15-69 (73,167,113) based on the data of the World Bank, and the proportion of males being MSM (3.4%) and TGW (0.68%) based on the evaluation of DataFolha Research Institute to obtain the number of MSM (2,487,682) and TGW (497,536) in Brazil^8^. Then using the proportion of suitable for PrEP (40.7%) and uptake rate of PrEP among MSM (15.4%) and TGW (4.3%) to obtain the PrEP users among MSM (151,689) and TGW (8,707) in Brazil^8-10^.

**Table S1 General parameters used in model and cost-effective analysis among MSM/TGW in all three countries**

| **Variable** | **Base-case** | **Range** | **Distribution** | **Ref.** |
| --- | --- | --- | --- | --- |
| ***Characteristics of STI transmission and progress*** |  |  |  |  |
| Probability of transmission of gonorrhoea, per partner | 0.301 | 0.11-0.60 | Beta (6.03, 14.01) | ^11-13^ |
| Probability of transmission of chlamydia, per partner | 0.346 | 0.11-0.67 | Beta (7.48, 14.14) | ^13-15^ |
| Probability of transmission of syphilis, per partner | 0.3 | 0.22-0.61 | Beta (5.99, 13.99) | ^16,17^ |
| Rate of natural recovery, NG, per year | 3.08 | 1.6-6.03 | Triangular (1.6, 3.1, 6.0) | ^18^ |
| Rate of natural recovery, CT, per year | 1.43 | 1.0-10.8 | Triangular (1.0, 1.4, 10.8) | ^13,14,19^ |
| Transition probability of syphilis, per month |  |  |  |  |
| from primary to secondary syphilis | 0.483 | … | … | ^20^ |
| from secondary to early latent syphilis | 0.237 | … | … | ^20^ |
| from early latent to late latent syphilis | 0.259 | … | … | ^20^ |
| from late latent to tertiary syphilis | 0.027 | … | … | ^20^ |
| Probability of recovery after treatment, NG, per month | 0.989 | … | … | ^18^ |
| Probability of recovery after treatment, CT, per month | 0.91 | … | … | ^14^ |
| Probability of recovery after treatment, syphilis, per month | 0.989 | … | … | ^18^ |
|  |  |  |  |  |
| ***Characteristics of STI epidemic*** |  |  |  |  |
| Hazard ratio for HIV infection with gonorrhoea co-infection | 3.4 | 2.1-5.2 | Triangular (2.1, 3.4, 5.2) | ^21^ |
| Hazard ratio for HIV infection with chlamydia co-infection | 2.6 | 1.7-3.7 | Triangular (1.7, 2.6, 3.7) | ^21^ |
| Hazard ratio for HIV infection with syphilis co-infection | 2.46 | 1.05-5.74 | Triangular (1.1, 2.5, 5.7) | ^22^ |
| Proportion of syphilis in different stages |  |  |  |  |
| Proportion of primary syphilis | 43.0% | … | … | ^23^ |
| Proportion of secondary syphilis | 16.0% | … | … | ^23^ |
| Proportion of early latent syphilis | 7.0% | … | … | ^23^ |
| Proportion of late latent syphilis | 34.0% | … | … | ^23^ |
| Proportion of tertiary syphilis | 0 | … | … | ^23^ |
|  |  |  |  |  |
| ***Characteristics of STI testing*** |  |  |  |  |
| Sensitivity for syphilis testing |  |  |  |  |
| Sensitivity for syphilis screening, primary | 0.86 | … | … | ^24^ |
| Sensitivity for syphilis screening, secondary | 1 | … | … | ^24^ |
| Sensitivity for syphilis screening, early latent | 0.98 | … | … | ^24^ |
| Sensitivity for syphilis screening, late latent | 0.73 | … | … | ^24^ |
| Sensitivity for syphilis screening, tertiary | 0.73 | … | … | ^24^ |
|  |  |  |  |  |
| ***Characteristics of health utility*** |  |  |  |  |
| Utility of NG infection, symptomatic | 0.84 | 0.76-0.92 | Beta (10.45, 1.99) | ^25,26^ |
| Utility of NG infection, asymptomatic | 1 | … | … | ^25^ |
| Utility of CT infection, symptomatic | 0.93 | 0.65-1 | Beta (23.29, 1.75) | ^27^ |
| Utility of chlamydia infection, asymptomatic | 1 | … | … | ^27^ |
| Utility of primary syphilis infection | 0.985 | 0.985-0.986 | Beta (144.55, 2.20) | ^28^ |
| Utility of secondary syphilis infection | 0.952 | 0.952-0.956 | Beta (434.07, 21.88) | ^28^ |
| Utility of latent syphilis infection | 0.952 | 0.952-0.956 | Beta (434.07, 21.88) | ^28^ |
| Utility of tertiary syphilis infection | 0.717 | 0.50-0.93 | Beta (13.83, 5.46) | ^28^ |
| Utility of HIV infection on ART | 0.94 | 0.66-1 | Beta (131.60, 8.40) | ^25^ |
|  |  |  |  |  |
| Discount rate, per year | 3% | 0-5% | Beta (8.7, 281.30) | ^29,30^ |

**Table S2** **Country-specific parameters used in model and cost-effective analysis among Australian MSM**

| **Variable** | **Base-case** | **Range** | **Distribution*** | **Ref.** |
| --- | --- | --- | --- | --- |
| ***Characteristics of population*** |  |  |  |  |
| Number of males, 0-14 | 2543515 | … | … | ^World bank 2020^ |
| Number of males, 15-64 | 8293954 | … | … | ^World bank 2020^ |
| Proportion of being MSM | 1.88% | 1.41%-6.04% | Beta (3.45, 180.02) | ^4,31^ |
| Rate of natural death, males, annually | 7.32% | … | … | ^World bank 2020^ |
|  |  |  |  |  |
| ***Characteristics of PrEP*** |  |  |  |  |
| Effectiveness of oral PrEP | 95% | 39%-99% | Beta (8.89, 13.90) | ^32,33^ |
| Proportion of MSM suitable for PrEP | 37.4% | … | … | ^5^ |
| Coverage of PrEP among MSM | 56.0% | … | … | ^34^ |
| Discontinuation rate of PrEP, per 100 PYs | 19.0 | 8.0-30.1 | Beta (2.73, 11.66) | ^35-37^ |
|  |  |  |  |  |
| ***Characteristics of STI transmission and progress*** |  |  |  |  |
| Proportion of sexual mixing among PrEP users |  |  |  |  |
| Have sex with PrEP users | 50.6% | … | … | ^38^ |
| Have sex with non-PrEP users | 49.4% | … | … | ^38^ |
| Proportion of sexual mixing among non-PrEP users |  |  |  |  |
| Have sex with PrEP users | 19.7% | … | … | ^38^ |
| Have sex with non-PrEP users | 80.3% | … | … | ^38^ |
|  |  |  |  |  |
| ***Characteristics of STI epidemic*** |  |  |  |  |
| HIV incidence among MSM, per 100 PYs | 1.87 | 1.40-2.49 | Beta (3.14, 179.09) | ^39^ |
| HIV mortality among males, per 100 PYs | 1.28 | 1.12-1.45 | Beta (1.60, 123.76) | ^40^ |
| Prevalence of NG among high-risk MSM | 11.1% | 7.6%-16.2% | Beta (4.27, 34.20) | ^41-43^ |
| Prevalence of NG among low-risk MSM | 4.0% | 2.4%-5.1% | Beta (15.32, 367.68) | ^41,44^ |
| Proportion of symptomatic infections, NG | 45% | 15%-89% | Beta (10.69, 13.06) | ^11,45,46^ |
| Prevalence of CT among high-risk MSM | 10.7% | 6.4%-16.1% | Beta (3.98, 33.24) | ^41-43^ |
| Prevalence of CT among low-risk MSM | 3.3% | 2.3%-5.8% | Beta (10.49, 307.61) | ^41,44^ |
| Proportion of symptomatic infections, CT | 26.6% | 11%-47% | Beta (4.93, 13.60) | ^45,47,48^ |
| Prevalence of syphilis among high-risk MSM | 7.0% | 6.6%-7.5% | Beta (1.75, 23.29) | ^41^ |
| Prevalence of syphilis among low-risk MSM | 2.4% | 2.3%-2.6% | Beta (5.60, 227.64) | ^41,44^ |
|  |  |  |  |  |
| ***Characteristics of Risk behaviours*** |  |  |  |  |
| Effectiveness of condom in preventing STI infection | 87.1% | 70%-95% | Beta (38.27, 5.67) | ^49^ |
| Coverage of consistent condom use, on PrEP | 64% | 60.7%-68.0% | Beta (14.11, 7.93) | ^50,51^ |
| Coverage of consistent condom use, not on PrEP | 84% | 78.8%-88.3% | Beta (44.32, 8.44) | ^50^ |
| Number of sex partners per month, PrEP users | 7.75 | ±30% | Triangular (5.4, 7.8, 10.1) | ^52^ |
| Number of sex partners per month, non-PrEP users | 2.33 | ±30% | Triangular (1.6, 2.3, 3.0) | ^52^ |
|  |  |  |  |  |
| ***Characteristics of STI testing*** |  |  |  |  |
| Average times of chlamydia testing for each non-PrEP user, per year | 3.22 times | … | … | ^42^ |
| Average times of gonorrhoea testing for each non-PrEP user, per year | 3.22 times | … | … | ^42^ |
| Average times of syphilis testing for each non-PrEP user, per year | 1.8 times | … | … | ^53,54^ |
|  |  |  |  |  |
| ***Characteristics of STI treatment*** |  |  |  |  |
| Probability of treatment due to symptoms, NG, per month | 0.094 | 0.023-0.12 | Beta (19.92, 192.00) | ^55,56^ |
| Probability of treatment due to symptoms, CT, per month | 0.094 | 0.023-0.12 | Beta (19.92, 192.00) | ^55,56^ |
| Coverage of NG treatment due to identification, per year | 88% | 62%-99% | Beta (36.29, 4.95) | ^53^ |
| Coverage of CT treatment due to identification, per year | 93% | 65%-99% | Beta (150.43, 11.32) | ^53^ |
| Coverage of syphilis treatment due to identification, per year | 89% | 62%-92% | Beta (33.96, 4.20) | ^57-59^ |
|  |  |  |  |  |
| ***Characteristics of costs, AUD*** |  |  |  |  |
| Oral PrEP drug, 30 pills | 42.5 | ±30% | Gamma (69.44, 1.63) | ^PBS^ |
| PrEP monitoring (HIV screening), per test | 15.65 | ±30% | Gamma (61.23, 3.91) | ^60^ |
| PrEP monitoring (HBV+HCV screening), per test | 34.5 | ±30% | Gamma (47.61, 1.38) | ^61^ |
| PrEP monitoring (serum testing), per test | 9.7 | ±30% | Gamma (94.09, 9.7) | ^61^ |
| PrEP monitoring (routine medical), per visit | 118.9 | ±30% | Gamma (72.13, 0.61) | ^61^ |
| NG testing, per test | 35.9 | ±30% | Gamma (7.63, 0.21) | ^MBS 69494^ |
| CT testing, per test | 28.7 | ±30% | Gamma (8.24, 0.29) | ^MBS 69316^ |
| Syphilis screening testing, per test | 5.64 | ±30% | Gamma (31.81, 5.64) | ^62^ |
| Syphilis confirmation testing, per test | 5.64 | ±30% | Gamma (31.81, 5.64) | ^62^ |
| HIV confirmation test, per test | 29.0 | ±30% | Gamma (52.56, 1.81) | ^60^ |
| HIV treatment, per treat per year | 15000.0 | 10000-20000 | Gamma (225.0, 0.02) | ^63^ |
| Gonorrhoea treatment, per treatment | 45.58 | ±30% | Gamma (57.71, 1.27) | ^PBS-1783W+8200N^ |
| Chlamydia treatment, per treatment | 22.31 | ±30% | Gamma (79.64, 3.57) | ^PBS-8200N^ |
| Syphilis treatment, per treatment | 309.22 | ±30% | Gamma (59.76, 0.19) | ^62^ |
| Medical consultation, per treatment | 36.0 | ±30% | Gamma (81.00, 2.25) | ^PBS-23^ |
|  |  |  |  |  |
| Willingness to pay (AUD) | 50,000 | … | … | ^64^ |

**Table S3 Country-specific parameters used in model and cost-effective analysis among Brazil MSM/TGW**

| **Variable** | **Base-case** | **Range** | **Distribution** | **Ref.** |
| --- | --- | --- | --- | --- |
| ***Characteristics of population*** |  |  |  |  |
| Number of males, 0-14 | 22,492,872 | … | … | ^World bank 2020^ |
| Number of males, 15-64 | 73,167,113 | … | … | ^World bank 2020^ |
| Proportion of being MSM | 3.4% | … | … | ^1^ |
| Proportion of being TGW | 0.34% | … | … | ^8^ |
|  |  |  |  |  |
| ***Characteristics of PrEP*** |  |  |  |  |
| Effectiveness of oral PrEP | 75% | 50%-95% | Beta (13.31, 4.43) | ^32,65^ |
| Proportion of MSM/TGW suitable for PrEP | 40.7% | 10%-80% | Beta (9.42, 13.72) | ^9^ |
| Coverage of PrEP among MSM/TGW | 15.4% | 4.3%-31% | Beta (7.87, 43.24) | ^10, PrEP Watch,66^ |
| Discontinuation rate of PrEP, per 100 PYs | 19.0 | 8.0-30.1 | Beta (11.51, 49.05) | ^35-37^ |
|  |  |  |  |  |
| ***Characteristics of STI transmission and progress for MSM/TGW*** |  |  |  |  |
| Proportion of sexual mixing among PrEP users |  |  |  |  |
| Have sex with PrEP users | 50.6% | … | … | ^38^ |
| Have sex with non-PrEP users | 49.4% | … | … | ^38^ |
| Proportion of sexual mixing among non-PrEP users |  |  |  |  |
| Have sex with PrEP users | 19.7% | … | … | ^38^ |
| Have sex with non-PrEP users | 80.3% | … | … | ^38^ |
|  |  |  |  |  |
| ***Characteristics of STI epidemic*** |  |  |  |  |
| HIV incidence among MSM, per 100 PYs | 6.65 | 5.17-8.39 | Beta (9.81, 141.13) | ^67^ |
| HIV incidence among TGW, per 100 PYs | 9.16 | 4.05-17.32 | Beta (8.38, 83.08) | ^67^ |
| HIV mortality among MSM/TGW, per 100 PYs | 7.1 | 5.0-9.2 | Beta (5.13, 67.16) | ^68^ |
| Prevalence of gonorrhoea among high-risk MSM | 14.6% | ±30% | Beta (45.36, 265.35) | ^44,69^ |
| Prevalence of gonorrhoea among low-risk MSM | 5.2% | ±30% | Beta (6.36, 115.88) | ^69^ |
| Prevalence of gonorrhoea among high-risk TGW | 23.0% | ±30% | Beta (16.06, 53.78) | ^44,70^ |
| Prevalence of gonorrhoea among low-risk TGW | 8.2% | 5%-15% | Beta (15.35, 171.84) | ^70^ |
| Proportion of symptomatic infections, NG | 13% | ±30% | Beta (36.63, 245.12) | ^71^ |
| Prevalence of chlamydia among high-risk MSM | 16.6% | ±30% | Beta (9.03, 45.35) | ^44,69^ |
| Prevalence of chlamydia among low-risk MSM | 5.2% | ±30% | Beta (6.36, 115.88) | ^69^ |
| Prevalence of chlamydia among high-risk TGW | 32.6% | ±30% | Beta (28.33, 58.56) | ^44,70^ |
| Prevalence of chlamydia among low-risk TGW | 10.2% | 5%-15% | Beta (3.64, 32.00) | ^70^ |
| Proportion of symptomatic infections, CT | 5% | ±30% | Beta (5.89, 111.86) | ^71^ |
| Prevalence of syphilis among high-risk MSM | 21.8% | ±30% | Beta (14.65, 52.54) | ^44,69^ |
| Prevalence of syphilis among low-risk MSM | 7.5% | 5%-15% | Beta (12.93, 159.50) | ^69^ |
| Prevalence of syphilis among high-risk TGW | 27.0% | 15%-30% | Beta (21.02, 56.82) | ^72^ |
| Prevalence of syphilis among low-risk TGW | 9.3% | ±30% | Beta (19.52, 190.36) | ^44,72^ |
|  |  |  |  |  |
| ***Characteristics of Risk behaviours*** |  |  |  |  |
| Effectiveness of condom in preventing STI infection | 87.1% | 70%-95% | Beta (38.27, 5.67) | ^49^ |
| Coverage of consistent condom use, MSM on PrEP | 48% | 34%-63% | Beta (32.80, 35.53) | ^TransOdara^ |
| Coverage of consistent condom use, TGW on PrEP | 19% | 13.3-24.7% | Beta (72.91, 310.84) | ^73^ |
| Coverage of consistent condom use, MSM not on PrEP | 63% | 44%-82% | Beta (22.32, 13.11) | ^TransOdara^ |
| Coverage of consistent condom use, TGW not on PrEP | 36.6% | 26%-48% | Beta (52.71, 91.31) | ^73^ |
| Number of sex partners per month, PrEP users | 2.77 | 1-5 | Triangular (1, 2.77, 5) | ^73,74^ |
| Number of sex partners per month, non-PrEP users | 1.25 | 0.33-1.67 | Triangular (0.33, 1.25, 1.67) | ^73,75^ |
|  |  |  |  |  |
| ***Characteristics of STI testing*** |  |  |  |  |
| Average times of chlamydia testing for each non-PrEP user, per year | 0.2 times | … | … | ^TransOdara^ |
| Average times of gonorrhoea testing for each non-PrEP user, per year | 0.2 times | … | … | ^TransOdara^ |
| Average times of syphilis testing for each non-PrEP user, per year | 0.7 times | … | … | ^TransOdara^ |
|  |  |  |  |  |
| ***Characteristics of STI treatment*** |  |  |  |  |
| Probability of treatment due to symptoms, NG | 90% | … | … | ^TransOdara^ |
| Probability of treatment due to symptoms, CT | 90% | … | … | ^TransOdara^ |
| Coverage of gonorrhoea treatment, per year | 88% | 62%-99% | Beta (18.08, 2.47) | ^53^ |
| Coverage of chlamydia treatment, per year | 93% | 65%-99% | Beta (23.29, 1.75) | ^53^ |
| Coverage of syphilis treatment (MSM), per year | 89% | 82%-92% | Beta (216.94, 26.81) | ^57-59^ |
| Coverage of syphilis treatment (TGW), per year | 31% | 25%-48% | Beta (26.21, 58.35) | ^76^ |
|  |  |  |  |  |
| ***Characteristics of costs, USD*** |  |  |  |  |
| Oral PrEP drug, 30 pills | 22.5 | ±30% | Gamma (69.44, 3.09) | ^77^ |
| PrEP monitoring (HIV screening), per test | 0.37 | ±30% | Gamma (85.56, 231.25) | [^www.aids.gov.br^](http://www.aids.gov.br)^.^ |
| HBV test, per test | 0.51 | ±30% | Gamma (72.25, 141.67) | ^TransOdara^ |
| HCV test, per test | 0.33 | ±30% | Gamma (68.06, 206.25) | ^TransOdara^ |
| PrEP monitoring (serum testing), per test | 0.69 | ±30% | Gamma (74.39, 107.81) | ^TransOdara^ |
| PrEP monitoring (routine medical), per visit | 3.73 | ±30% | Gamma (68.71, 18.42) | ^78,79^ |
| Gonorrhoea testing, 3 sites, per test | 10.2 | ±30% | Gamma (69.91, 6.85) | ^TransOdara^ |
| Chlamydia testing, 3 sites, per test | 10.2 | ±30% | Gamma (69.91, 6.85) | ^TransOdara^ |
| Syphilis testing, per test | 0.4 | ±30% | Gamma (64.0, 160.0) | ^TransOdara^ |
| Gonorrhoea treatment, per treatment | 2.85 | ±30% | Gamma (70.26, 24.65) | ^TransOdara^ |
| Chlamydia treatment, per treatment | 1.35 | ±30% | Gamma (71.19, 52.73) | ^TransOdara^ |
| Syphilis treatment, per treatment | 43.0 | ±30% | Gamma (69.44, 1.61) | ^TransOdara^ |
| HIV treatment, per treat per year | 1960.0 | ±30% | Gamma (15.37, 0.01) | ^78^ |
|  |  |  |  |  |
| Willingness to pay (USD) | 8,786 | … | … | ^80^ |

TransOdara: <https://www.nudhes.org/transodara>

**Table S4 Country-specific parameters used in model and cost-effective analysis among Thailand MSM/TGW**

| **Variable** | **Base-case** | **Range** | **Distribution** | **Ref.** |
| --- | --- | --- | --- | --- |
| ***Characteristics of population*** |  |  |  |  |
| Number of males, 0-14 | 5866729 | … | … | ^World bank 2020^ |
| Number of males, 15-64 | 24021659 | … | … | ^World bank 2020^ |
| Proportion of being MSM | 4.24% | 2.61-5.88% | Beta (17.65, 392.86) | ^6^ |
| Proportion of being TGW | 0.31% | … | … | ^6^ |
| Number of MSM who are sexually active | 647,600 | … | … | ^6^ |
| Rate of natural death, males, annually | 0.1845 | … | … | ^World bank 2020^ |
|  |  |  |  |  |
| ***Characteristics of PrEP*** |  |  |  |  |
| Effectiveness of oral PrEP | 75% | 50%-95% | Beta (16.61, 5.54) | ^32,65^ |
| Proportion of MSM suitable for PrEP | 20.5% | … | … | ^6,7^ |
| Proportion of TGW suitable for PrEP | 48.2% | 38.7%-57.9% | Beta (74.73, 80.31) | ^6^ |
| Coverage of PrEP among MSM/TGW | 31.0% | 10%-60% | Beta (6.32, 14.07) | ^6, PrEPWatch^ |
| Discontinuation rate of PrEP, per 100 PYs | 19.0 | 8.0-30.1 | Beta (18.09, 77.10) | ^35-37^ |
|  |  |  |  |  |
| ***Characteristics of STI transmission and progress for MSM/TGW*** |  |  |  |  |
| Proportion of sexual mixing among PrEP users |  |  |  |  |
| Have sex with PrEP users | 50.6% | … | … | ^38^ |
| Have sex with non-PrEP users | 49.4% | … | … | ^38^ |
| Proportion of sexual mixing among non-PrEP users |  |  |  |  |
| Have sex with PrEP users | 19.7% | … | … | ^38^ |
| Have sex with non-PrEP users | 80.3% | … | … | ^38^ |
|  |  |  |  |  |
| ***Characteristics of STI epidemic*** |  |  |  |  |
| HIV incidence among MSM, per 100 PYs | 3.5 | 2.5-4.8 | Beta (11.79, 324.96) | ^81^ |
| HIV incidence among TGW, per 100 PYs | 4.5 | 1.5-5.3 | Beta (19.29, 409.46) | ^82^ |
| HIV mortality among males, per 100 PYs | 4.8 | 4.7-4.9 | Beta (21.88, 434.07) | ^83^ |
| Prevalence of NG among high-risk MSM | 15.5% | 6.4%-20.2% | Beta (22.40, 122.13) | ^84,85^ |
| Prevalence of NG among low-risk MSM | 5.5% | 2.3%-7.2% | Beta (28.53, 490.22) | ^44,85^ |
| Prevalence of NG among high-risk TGW | 13.1% | ±30% | Beta (37.15, 246.45) | ^86^ |
| Prevalence of NG among low-risk TGW | 4.7% | ±30% | Beta (5.22, 105.76) | ^44,86^ |
| Proportion of symptomatic infections, NG | 2.8% | 1%-5% | Beta (7.59, 263.57) | ^84^ |
| Prevalence of CT among high-risk MSM | 21.7% | 14.3%-23% | Beta (91.96, 331.82) | ^84,85^ |
| Prevalence of CT among low-risk MSM | 6.8% | 4.5%-7.2% | Beta (43.03, 589.73) | ^44,85^ |
| Prevalence of CT among high-risk TGW | 21.3% | ±30% | Beta (89.05, 329.03) | ^86^ |
| Prevalence of CT among low-risk TGW | 6.7% | ±30% | Beta (10.40, 144.87) | ^44,86^ |
| Proportion of symptomatic infections, CT | 2.8% | 1%-5% | Beta (7.59, 263.57) | ^84^ |
| Prevalence of syphilis among high-risk MSM | 9.8% | 6.9%-38.4% | Beta (2.31, 21.25) | ^87,88^ |
| Prevalence of syphilis among low-risk MSM | 3.4% | 2.4%-13.2% | Beta (2.76, 78.35) | ^44,89^ |
| Prevalence of syphilis among high-risk TGW | 38.4% | ±30% | Beta (226.69, 363.66) | ^87^ |
| Prevalence of syphilis among low-risk TGW | 13.2% | ±30% | Beta (37.68, 247.76) | ^44,87^ |
|  |  |  |  |  |
| ***Characteristics of Risk behaviours*** |  |  |  |  |
| Effectiveness of condom in preventing STI infection | 87.1% | 70%-95% | Beta (38.27, 5.67) | ^49^ |
| Coverage of consistent condom use, MSM on PrEP | 51.4% | 21.5%-84% | Beta (7.08, 6.69) | ^7,90^ |
| Coverage of consistent condom use, TGW on PrEP | 21.1% | ±30% | Beta (3.30, 12.35) | ^90^ |
| Coverage of consistent condom use, MSM not on PrEP | 70.8% | 14%-84% | Beta (6.76, 2.79) | ^7,50,91^ |
| Coverage of consistent condom use, TGW not on PrEP | 21.5% | ±30% | Beta (3.41, 12.46) | ^90^ |
| Number of sex partners per month, PrEP users | 3.5 | 1-5 | Triangular (1, 3.5, 5) | ^92,93^ |
| Number of sex partners per month, non-PrEP users | 1 | 0.5-2.5 | Triangular (0.5, 1, 2.5) | ^93^ |
|  |  |  |  |  |
| ***Characteristics of STI testing*** |  |  |  |  |
| Average times of chlamydia testing for each non-PrEP user, per year | 0.398 | … | … | ^94^ |
| Average times of gonorrhoea testing for each non-PrEP user, per year | 0.398 | … | … | ^94^ |
| Average times of syphilis testing for each non-PrEP user, per year | 0.398 | … | … | ^94^ |
|  |  |  |  |  |
| ***Characteristics of STI treatment*** |  |  |  |  |
| probability of treatment due to symptoms, NG, last 3 month | 67.4% | 47%-88% | Beta (14.14, 6.84) | ^95^ |
| probability of treatment due to symptoms, CT, last 3 month | 67.4% | 47%-88% | Beta (14.14, 6.84) | ^95^ |
| Coverage of NG treatment due to identification, per year | 88% | 62%-99% | Beta (18.08, 2.47) | ^53^ |
| Coverage of CT treatment due to identification, per year | 93% | 65%-99% | Beta (150.43, 11.32) | ^53^ |
| Coverage of syphilis treatment due to identification, per year | 89% | 82%-92% | Beta (216.94, 26.81) | ^57-59^ |
|  |  |  |  |  |
| ***Characteristics of costs, THB*** |  |  |  |  |
| Oral PrEP drug, 30 pills | 550.0 | ±30% | Gamma (69.44, 0.13) | ^65,PULSE^ |
| PrEP monitoring (HIV screening), per test | 200.0 | ±30% | Gamma (69.44, 0.35) | ^96, Insti^ |
| Monitoring on PrEP for initiation, one time | 918.0 | ±30% | Gamma (69.65, 0.08) | ^65,96^ |
| Monitoring on PrEP for follow-up, each year |  |  |  |  |
| Personnel, per year | 873.5 | ±30% | Gamma (63.06, 0.07) | ^65^ |
| HIV test (screening), per test | 200.0 | ±30% | Gamma (69.44, 0.35) | ^96, Insti^ |
| HIV test (confirmation), per test | 400.0 | ±30% | Gamma (64.00, 0.16) | ^96, Insti^ |
| STI test (chlamydia, gonorrhoea, syphilis rapid test and NAAT), per test | 550.0 | ±30% | Gamma (69.45, 0.13) | ^65^ |
| NG testing, per test | 750.0 | ±30% | Gamma (69.44, 0.09) | ^Medconsult clinic^ |
| Number of testing sites of NG test | 3 | … | … |  |
| CT testing, per test | 750.0 | ±30% | Gamma (69.44, 0.09) | ^Medconsult clinic^ |
| Number of testing sites of CT test | 3 | … | … |  |
| Syphilis screening testing, per test (VDRL) | 240.0 | ±30% | Gamma (64.00, 0.27) | ^Medconsult clinic^ |
| Syphilis confirmation testing, per test | 240.0 | ±30% | Gamma (64.00, 0.27) | ^Medconsult clinic^ |
| NG treatment, per treatment | 202.0 | ±30% | Gamma (45.34, 0.22) | ^Insti^ |
| CT treatment, per treatment | 170.0 | ±30% | Gamma (72.25, 0.43) | ^Insti^ |
| Syphilis treatment, per treatment | 1260.0 | ±30% | Gamma (70.56, 0.06) | ^Insti^ |
| HIV treatment, per treat per year | 12394.0 | ±30% | Gamma (153.61, 0.01) | ^96^ |
|  |  |  |  |  |
| Willingness to pay (THB) | 160,000 | … | … | ^97,98^ |

PrEP Watch: <https://www.prepwatch.org/>; PULSE: <https://www.pulse-clinic.com/buypreponline>; INSTI: <https://thailandhivtest.com/hiv-test-price/>; Medconsult clinic: <https://www.medconsultasia.com/services/sexual-health-testing/>

**Reference**

1. Sirijampa A, Chinviriyasit S, Chinviriyasit W. Hopf bifurcation analysis of a delayed SEIR epidemic model with infectious force in latent and infected period. *Adv Differ Equ* 2018; **2018**(1): 348.

2. Rafique M, Rehamn MAU, Alqahtani AM, et al. A new epidemic model of sexually transmittable diseases: a fractional numerical approach. *Scientific Reports* 2025; **15**(1): 3784.

3. Reisner SL, Choi SK, Herman JL, Bockting W, Krueger EA, Meyer IH. Sexual orientation in transgender adults in the United States. *BMC public health* 2023; **23**(1): 1799.

4. Zablotska IB, Gray R, Whittaker B, et al. The estimated number of potential PrEP users among gay-identifying men who have sex with men in Australia. *PloS one* 2018; **13**(10): e0204138.

5. society TKIfiaii. HIV, viral hepatitis and sexually transmissible infections in Australia Annual surveillance report 2021. Sydney, 2021.

6. Jacobson J. Estimation of PrEP Targets for Key and High-Risk Populations in Thailand, 2020-2022: UNAIDS, 2019.

7. Ramautarsing RA, Meksena R, Sungsing T, et al. Evaluation of a pre-exposure prophylaxis programme for men who have sex with men and transgender women in Thailand: learning through the HIV prevention cascade lens. *Journal of the International AIDS Society* 2020; **23 Suppl 3**(Suppl 3): e25540.

8. Spizzirri G, Eufrásio R, Abdo CHN, Lima MCP. Proportion of ALGBT adult Brazilians, sociodemographic characteristics, and self-reported violence. *Sci Rep* 2022; **12**(1): 11176.

9. Torres TS, Nascimento AR, Coelho LE, et al. Preferences for PrEP modalities among gay, bisexual, and other men who have sex with men from Brazil, Mexico, and Peru: a cross-sectional study. *Ther Adv Infect Dis* 2023; **10**: 20499361231153548.

10. Kerr LR. MSM in Brazil: baseline national data for prevalence of HIV; 2010.

11. Whittles LK, Didelot X, White PJ. Public health impact and cost-effectiveness of gonorrhoea vaccination: an integrated transmission-dynamic health-economic modelling analysis. *The Lancet Infectious diseases* 2022.

12. Garnett GP, Mertz KJ, Finelli L, Levine WC, St Louis ME. The transmission dynamics of gonorrhoea: modelling the reported behaviour of infected patients from Newark, New Jersey. *Philos Trans R Soc Lond B Biol Sci* 1999; **354**(1384): 787-97.

13. Azizi A, Ríos-Soto K, Mubayi A, J MH. A Risk-based Model for Predicting the Impact of using Condoms on the Spread of Sexually Transmitted Infections. *Infect Dis Model* 2017; **2**(1): 100-12.

14. Looker KJ, Wallace LA, Turner KM. Impact and cost-effectiveness of chlamydia testing in Scotland: a mathematical modelling study. *Theor Biol Med Model* 2015; **12**: 2.

15. Lewis J, White PJ, Price MJ. Per-partnership transmission probabilities for Chlamydia trachomatis infection: evidence synthesis of population-based survey data. *Int J Epidemiol* 2021; **50**(2): 510-7.

16. Towns JM, Huffam S, Chow EPF, et al. Clinical factors associated with syphilis concordance in men in sexual partnerships: a cross-sectional couples study. *Sexually transmitted infections* 2018; **94**(8): 571-3.

17. Castillo-Laborde C, Gajardo P, Nájera-De Ferrari M, et al. Modelling cost-effectiveness of syphilis detection strategies in prisoners: exploratory exercise in a Chilean male prison. *Cost Eff Resour Alloc* 2021; **19**(1): 5.

18. Whittles LK, Didelot X, White PJ. Public health impact and cost-effectiveness of gonorrhoea vaccination: an integrated transmission-dynamic health-economic modelling analysis. *The Lancet Infectious diseases* 2022; **22**(7): 1030-41.

19. Kretzschmar M, Welte R, van den Hoek A, Postma MJ. Comparative model-based analysis of screening programs for Chlamydia trachomatis infections. *Am J Epidemiol* 2001; **153**(1): 90-101.

20. Okuonghae D, Gumel AB, Ikhimwin BO, Iboi E. Mathematical Assessment of the Role of Early Latent Infections and Targeted Control Strategies on Syphilis Transmission Dynamics. *Acta Biotheor* 2019; **67**(1): 47-84.

21. Cheung KT, Fairley CK, Read TR, et al. HIV Incidence and Predictors of Incident HIV among Men Who Have Sex with Men Attending a Sexual Health Clinic in Melbourne, Australia. *PloS one* 2016; **11**(5): e0156160.

22. Guy RJ, Spelman T, Stoove M, et al. Risk factors for HIV seroconversion in men who have sex with men in Victoria, Australia: results from a sentinel surveillance system. *Sexual health* 2011; **8**(3): 319-29.

23. Forrest CE, Ward A. Clinical diagnosis of syphilis: a ten-year retrospective analysis in a South Australian urban sexual health clinic. *International journal of STD & AIDS* 2016; **27**(14): 1334-7.

24. Larsen SA, Steiner BM, Rudolph AH. Laboratory diagnosis and interpretation of tests for syphilis. *Clin Microbiol Rev* 1995; **8**(1): 1-21.

25. Reitsema M, Steffers L, Visser M, et al. Cost-effectiveness of increased HIV testing among MSM in The Netherlands. *AIDS (London, England)* 2019; **33**(12): 1807-17.

26. Li Y, Rönn MM, Tuite AR, et al. Estimated costs and quality-adjusted life-years lost due to N. gonorrhoeae infections acquired in 2015 in the United States: A modelling study of overall burden and disparities by age, race/ethnicity, and other factors. *Lancet Reg Health Am* 2022; **16**: 100364.

27. Huntington SE, Burns RM, Harding-Esch E, et al. Modelling-based evaluation of the costs, benefits and cost-effectiveness of multipathogen point-of-care tests for sexually transmitted infections in symptomatic genitourinary medicine clinic attendees. *BMJ Open* 2018; **8**(9): e020394.

28. Lopez ADM, Colin D.; Ezzati, Majid; Jamison, Dean T.; Murray, Christopher J. L.. . Global Burden of Disease and Risk Factors. Washington, DC: World Bank and Oxford University Press, 2006.

29. WHO. WHO guide to cost-effectiveness analysis. Geneva: World Health Organization, 2003.

30. Foundation BMG. Methods for economic evaluation project (MEEP): the Gates reference case. Seattle, WA: Bill & Melinda Gates Foundation, 2014.

31. Wilson T, Temple J, Lyons A, Shalley F. What is the size of Australia's sexual minority population? *BMC Res Notes* 2020; **13**(1): 535.

32. Riddell Jt, Amico KR, Mayer KH. HIV Preexposure Prophylaxis: A Review. *Jama* 2018; **319**(12): 1261-8.

33. Schneider K, Gray RT, Wilson DP. A cost-effectiveness analysis of HIV preexposure prophylaxis for men who have sex with men in Australia. *Clinical infectious diseases : an official publication of the Infectious Diseases Society of America* 2014; **58**(7): 1027-34.

34. Holt M, Broady TR, Mao L, et al. Increasing preexposure prophylaxis use and 'net prevention coverage' in behavioural surveillance of Australian gay and bisexual men. *AIDS (London, England)* 2021; **35**(5): 835-40.

35. Jin F, Amin J, Guy R, et al. Adherence to daily HIV Pre-exposure prophylaxis in a large-scale implementation study in New South Wales, Australia. *AIDS (London, England)* 2021.

36. Vaccher SJ, Marzinke MA, Templeton DJ, et al. Predictors of Daily Adherence to HIV Pre-exposure Prophylaxis in Gay/Bisexual Men in the PRELUDE Demonstration Project. *AIDS and behavior* 2019; **23**(5): 1287-96.

37. Coyer L, van den Elshout MAM, Achterbergh RCA, et al. Understanding pre-exposure prophylaxis (PrEP) regimen use: Switching and discontinuing daily and event-driven PrEP among men who have sex with men. *EClinicalMedicine* 2020; **29-30**: 100650.

38. Wang L, Moqueet N, Lambert G, et al. Population-Level Sexual Mixing According to HIV Status and Preexposure Prophylaxis Use Among Men Who Have Sex With Men in Montreal, Canada: Implications for HIV Prevention. *Am J Epidemiol* 2020; **189**(1): 44-54.

39. Selvey LA, Slimings C, Adams E, Manuel J. Incidence and predictors of HIV, chlamydia and gonorrhoea among men who have sex with men attending a peer-based clinic. *Sexual health* 2018; **15**(5): 451-9.

40. Institute K. Australian HIV Observational Database Annual Report 2016: Sydney: Kirby Institute, UNSW Australia, 2016.

41. Martín-Sánchez M, Case R, Fairley C, et al. Trends and differences in sexual practices and sexually transmitted infections in men who have sex with men only (MSMO) and men who have sex with men and women (MSMW): a repeated cross-sectional study in Melbourne, Australia. *BMJ Open* 2020; **10**(11): e037608.

42. McManus H, Grulich AE, Amin J, et al. Comparison of Trends in Rates of Sexually Transmitted Infections Before vs After Initiation of HIV Preexposure Prophylaxis Among Men Who Have Sex With Men. *JAMA Netw Open* 2020; **3**(12): e2030806.

43. Ong JJ, Baggaley RC, Wi TE, et al. Global Epidemiologic Characteristics of Sexually Transmitted Infections Among Individuals Using Preexposure Prophylaxis for the Prevention of HIV Infection: A Systematic Review and Meta-analysis. *JAMA Netw Open* 2019; **2**(12): e1917134.

44. Montaño MA, Dombrowski JC, Dasgupta S, et al. Differences in sexually transmitted infection risk comparing preexposure prophylaxis users and propensity score matched historical controls in a clinic setting. *AIDS (London, England)* 2019; **33**(11): 1773-80.

45. Korenromp EL, Sudaryo MK, de Vlas SJ, et al. What proportion of episodes of gonorrhoea and chlamydia becomes symptomatic? *International journal of STD & AIDS* 2002; **13**(2): 91-101.

46. Ong JJ, Fethers K, Howden BP, et al. Asymptomatic and symptomatic urethral gonorrhoea in men who have sex with men attending a sexual health service. *Clin Microbiol Infect* 2017; **23**(8): 555-9.

47. Yeung AH, Temple-Smith M, Fairley CK, et al. Chlamydia prevalence in young attenders of rural and regional primary care services in Australia: a cross-sectional survey. *Med J Aust* 2014; **200**(3): 170-5.

48. Tuddenham S, Hamill MM, Ghanem KG. Diagnosis and Treatment of Sexually Transmitted Infections: A Review. *Jama* 2022; **327**(2): 161-72.

49. Remis RS, Alary M, Liu J, Kaul R, Palmer RW. HIV transmission among men who have sex with men due to condom failure. *PloS one* 2014; **9**(9): e107540.

50. Hardy C, Fairley CK, Ong JJ, et al. Drug and Alcohol Use with Condomless Anal Sex among Men Who Have Sex with Men in Melbourne, Australia: A Retrospective Data Analysis from 2011 to 2017. *Arch Sex Behav* 2021.

51. Lal L, Audsley J, Murphy DA, et al. Medication adherence, condom use and sexually transmitted infections in Australian preexposure prophylaxis users. *AIDS (London, England)* 2017; **31**(12): 1709-14.

52. Keen P, Hammoud MA, Bourne A, et al. Use of HIV Pre-exposure Prophylaxis (PrEP) Associated With Lower HIV Anxiety Among Gay and Bisexual Men in Australia Who Are at High Risk of HIV Infection: Results From the Flux Study. *Journal of acquired immune deficiency syndromes (1999)* 2020; **83**(2): 119-25.

53. Institute TK. National update on HIV, viral hepatitis and sexually transmissible infections in Australia, 2009-2018. Australia, 2020.

54. Chow EPF, Callander D, Fairley CK, et al. Increased Syphilis Testing of Men Who Have Sex With Men: Greater Detection of Asymptomatic Early Syphilis and Relative Reduction in Secondary Syphilis. *Clinical infectious diseases : an official publication of the Infectious Diseases Society of America* 2017; **65**(3): 389-95.

55. Ong JJ, Ruan L, Lim AG, et al. Impact of screening on the prevalence and incidence of Mycoplasma genitalium and its macrolide resistance in men who have sex with men living in Australia: A mathematical model. *EClinicalMedicine* 2021; **33**: 100779.

56. Zhang L, Regan DG, Chow EPF, et al. Neisseria gonorrhoeae Transmission Among Men Who Have Sex With Men: An Anatomical Site-Specific Mathematical Model Evaluating the Potential Preventive Impact of Mouthwash. *Sexually transmitted diseases* 2017; **44**(10): 586-92.

57. Keen P, Gray RT, Telfer B, et al. The 2016 HIV diagnosis and care cascade in New South Wales, Australia: meeting the UNAIDS 90-90-90 targets. *Journal of the International AIDS Society* 2018; **21**(4): e25109.

58. Marukutira T, Gray RT, Douglass C, et al. Gaps in the HIV diagnosis and care cascade for migrants in Australia, 2013-2017: A cross-sectional study. *PLoS medicine* 2020; **17**(3): e1003044.

59. UNAIDS. UNAIDS data 2020, 2020.

60. Williams OD, Dean JA, Crothers A, Gilks CF, Gow J. Economic evaluation of alternative testing regimes and settings to detect undiagnosed HIV in Australia. *BMC Health Serv Res* 2021; **21**(1): 30.

61. Schneider K, Gray RT, Wilson DP. A Cost-effectiveness Analysis of HIV Preexposure Prophylaxis for Men Who Have Sex With Men in Australia. *Clinical Infectious Diseases* 2014; **58**(7): 1027-34.

62. Jayawardena T, Hoad V, Styles C, et al. Modelling the risk of transfusion-transmitted syphilis: a reconsideration of blood donation testing strategies. *Vox Sang* 2019; **114**(2): 107-16.

63. Gray RT, Watson J, Cogle AJ, et al. Funding antiretroviral treatment for HIV-positive temporary residents in Australia prevents transmission and is inexpensive. *Sexual health* 2018; **15**(1): 13-9.

64. George B, Harris A, Mitchell A. Cost-effectiveness analysis and the consistency of decision making: evidence from pharmaceutical reimbursement in australia (1991 to 1996). *Pharmacoeconomics* 2001; **19**(11): 1103-9.

65. Suraratdecha C, Stuart RM, Manopaiboon C, et al. Cost and cost-effectiveness analysis of pre-exposure prophylaxis among men who have sex with men in two hospitals in Thailand. *Journal of the International AIDS Society* 2018; **21 Suppl 5**(Suppl Suppl 5): e25129.

66. Blair KJ, Torres TS, Hoagland B, et al. Pre-exposure prophylaxis use, HIV knowledge, and internalized homonegativity among men who have sex with men in Brazil: A cross-sectional study. *Lancet Reg Health Am* 2022; **6**.

67. Teixeira SL, Jalil CM, Jalil EM, et al. Evidence of an untamed HIV epidemic among MSM and TGW in Rio de Janeiro, Brazil: a 2018 to 2020 cross-sectional study using recent infection testing. *Journal of the International AIDS Society* 2021; **24**(6): e25743.

68. Saúde SdVe. Boletim Epidemiológico - HIV/Aids 2021, 2021.

69. Cunha CB, Friedman RK, de Boni RB, et al. Chlamydia trachomatis, Neisseria gonorrhoeae and syphilis among men who have sex with men in Brazil. *BMC public health* 2015; **15**: 686.

70. Paiatto BNM, Mayaud P, Araújo S, et al. O15.5 Prevalence and risk factors for chlamydia and gonorrhea among transgender women in 2019–20 in São Paulo, Brazil. *Sexually transmitted infections* 2021; **97**(Suppl 1): A51-A2.

71. Moreira JS, Vasconcelos R, Doi AM, Avelino-Silva VI. Real-life occurrence of bacterial sexually transmitted infections among PrEP users: improving the diagnosis of Chlamydia trachomatis and Neisseria gonorrhoeae with multisite screening. *Rev Inst Med Trop Sao Paulo* 2021; **63**: e76.

72. Fernandes FR, Zanini PB, Rezende GR, et al. Syphilis infection, sexual practices and bisexual behaviour among men who have sex with men and transgender women: a cross-sectional study. *Sexually transmitted infections* 2015; **91**(2): 142-9.

73. Jalil EM, Torres TS, de APCC, et al. High Rates of Sexualized Drug Use or Chemsex among Brazilian Transgender Women and Young Sexual and Gender Minorities. *International journal of environmental research and public health* 2022; **19**(3).

74. Grinsztejn B, Hoagland B, Moreira RI, et al. Retention, engagement, and adherence to pre-exposure prophylaxis for men who have sex with men and transgender women in PrEP Brasil: 48 week results of a demonstration study. *The lancet HIV* 2018; **5**(3): e136-e45.

75. Brignol SM, Dourado I, Amorim LD, Miranda JG, Kerr LR. Social networks of men who have sex with men: a study of recruitment chains using Respondent Driven Sampling in Salvador, Bahia State, Brazil. *Cad Saude Publica* 2015; **31 Suppl 1**: 170-81.

76. Bassichetto K, Dourado I, Magno L, et al. P409 Testing and treating syphilis in transgender women – a point-of-care approach in Brazil. *Sexually transmitted infections* 2021; **97**(Suppl 1): A167-A.

77. S Md. Contract Number 140/2017 Betweeen the Brazilian Ministry of Health and Gilead Sciences Farmceuticals.; 2017.

78. Araújo DV, Bahia L, Stella SF. The Economic Burden of HIV/AIDS and myocardial infarction treatment in Brazil. *ScientificWorldJournal* 2013; **2013**: 864962.

79. De Boni RB, Machado IK, De Vasconcellos MTL, et al. Syndemics among individuals enrolled in the PrEP Brasil Study. *Drug and Alcohol Dependence* 2018; **185**: 168-72.

80. Nsengiyumva NP, Campbell JR, Oxlade O, et al. Scaling up target regimens for tuberculosis preventive treatment in Brazil and South Africa: An analysis of costs and cost-effectiveness. *PLoS medicine* 2022; **19**(6): e1004032.

81. Kritsanavarin U, Bloss E, Manopaiboon C, et al. HIV incidence among men who have sex with men and transgender women in four provinces in Thailand. *International journal of STD & AIDS* 2020; **31**(12): 1154-60.

82. van Griensven F, Phanuphak N, Manopaiboon C, et al. HIV prevalence and incidence among men who have sex with men and transgender women in Bangkok, 2014-2018: Outcomes of a consensus development initiative. *PloS one* 2022; **17**(1): e0262694.

83. Teeraananchai S, Kerr SJ, Ruxrungtham K, et al. Loss to follow-up and associated factors of patients in the National AIDS Program in Thailand. *Antiviral therapy* 2018; **23**(6): 529-38.

84. Pattanasin S, Dunne EF, Wasinrapee P, et al. Screening for Chlamydia trachomatis and Neisseria gonorrhoeae infection among asymptomatic men who have sex with men in Bangkok, Thailand. *International journal of STD & AIDS* 2018; **29**(6): 577-87.

85. Hiransuthikul A, Sungsing T, Jantarapakde J, et al. Correlations of chlamydia and gonorrhoea among pharyngeal, rectal and urethral sites among Thai men who have sex with men: multicentre community-led test and treat cohort in Thailand. *BMJ Open* 2019; **9**(6): e028162.

86. Hiransuthikul A, Janamnuaysook R, Sungsing T, et al. High burden of chlamydia and gonorrhoea in pharyngeal, rectal and urethral sites among Thai transgender women: implications for anatomical site selection for the screening of STI. *Sexually transmitted infections* 2019; **95**(7): 534-9.

87. Pattanasin S, Griensven FV, Mock PA, et al. HIV and syphilis prevalence among transgender women and men who have sex with men, Silom Community Clinic, Bangkok, Thailand, 2017-2019. *AIDS care* 2022; **34**(10): 1305-13.

88. (CDC). CfDCaP. HIV and syphilis infection among men who have sex with men--Bangkok, Thailand, 2005-2011. *MMWR Morb Mortal Wkly Rep* 2013; **62**(25): 518-20.

89. (CDC) CfDCaP. HIV and syphilis infection among men who have sex with men--Bangkok, Thailand, 2005-2011. *MMWR Morb Mortal Wkly Rep* 2013; **62**(25): 518-20.

90. Seekaew P, Pengnonyang S, Jantarapakde J, et al. Characteristics and HIV epidemiologic profiles of men who have sex with men and transgender women in key population-led test and treat cohorts in Thailand. *PloS one* 2018; **13**(8): e0203294.

91. Buchbinder SP, Glidden DV, Liu AY, et al. HIV pre-exposure prophylaxis in men who have sex with men and transgender women: a secondary analysis of a phase 3 randomised controlled efficacy trial. *The Lancet Infectious diseases* 2014; **14**(6): 468-75.

92. Velloza J, Bacchetti P, Hendrix CW, et al. Short- and Long-Term Pharmacologic Measures of HIV Pre-exposure Prophylaxis Use Among High-Risk Men Who Have Sex With Men in HPTN 067/ADAPT. *Journal of acquired immune deficiency syndromes (1999)* 2019; **82**(2): 149-58.

93. Liu AY, Norwood A, Gundacker H, et al. Brief Report: Routine Use of Oral PrEP in a Phase 2 Rectal Microbicide Study of Tenofovir Reduced-Glycerin 1% Gel (MTN-017). *Journal of acquired immune deficiency syndromes (1999)* 2019; **81**(5): 516-20.

94. Thepthien BO, Srivanichakorn S, Udomsubpayakul U, Sein Win ZZK, Zaw AMM. HIV risk behavior and testing among MS M in Bangkok 2015-2019: a short report. *AIDS care* 2022; **34**(7): 869-77.

95. Phrasisombath K, Thomsen S, Sychareun V, Faxelid E. Care seeking behaviour and barriers to accessing services for sexually transmitted infections among female sex workers in Laos: a cross-sectional study. *BMC Health Serv Res* 2012; **12**: 37.

96. Leelahavarong P, Teerawattananon Y, Werayingyong P, et al. Is a HIV vaccine a viable option and at what price? An economic evaluation of adding HIV vaccination into existing prevention programs in Thailand. *BMC public health* 2011; **11**: 534.

97. Committee. NLoEM. Sub-committee of Thai Working Group on Health Technology Assessment 2013 meeting report of 2nd annual meeting, 2013.

98. Nimdet K, Ngorsuraches S. Willingness to pay per quality-adjusted life year for life-saving treatments in Thailand. *BMJ Open* 2015; **5**(10): e008123.

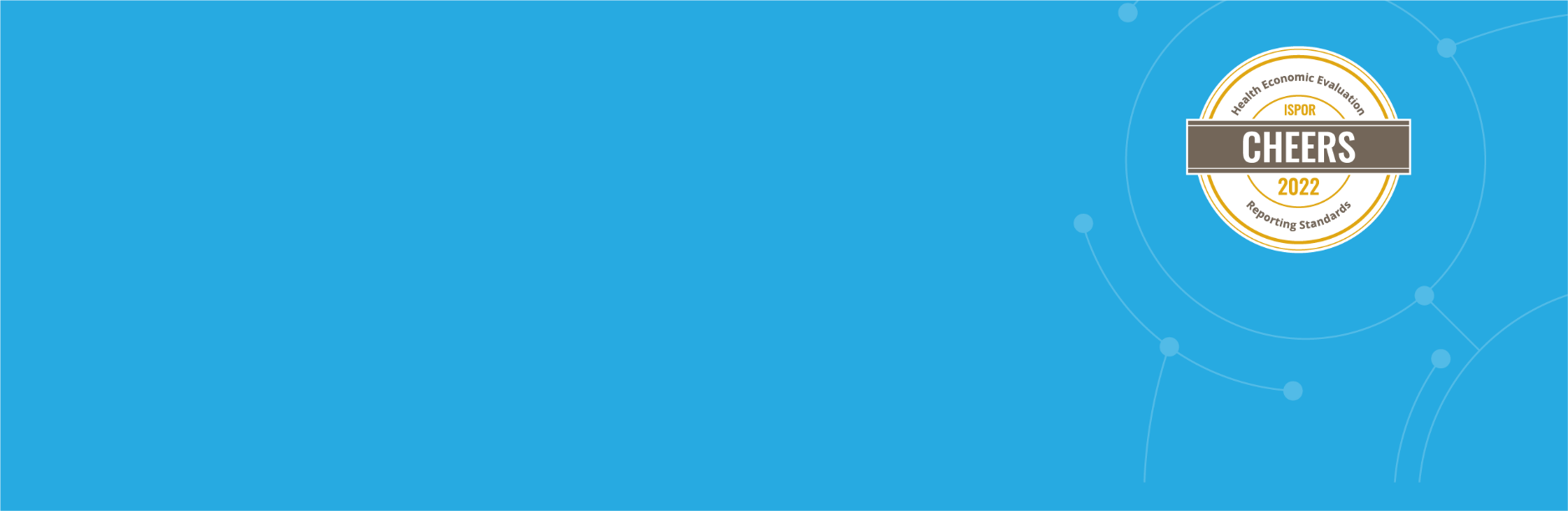


**Consolidated Health Economic Evaluation Reporting Standards
(CHEERS) 2022 Checklist**

The CHEERS 2022 statement replaces the 2013 CHEERS statement, which should no longer
be used. The CHEERS 2022 checklist contains 28 items with accompanying descriptions.
Checklist users should indicate the section of the manuscript where relevant information
can be found. The authors recommend using a section heading with a paragraph number.
If an item does not apply to a particular economic evaluation, checklist users are encouraged
to report “Not Applicable.” If information is otherwise not reported, checklist users are encouraged to

write, “Not Reported.” Users should avoid the term “Not Conducted” as CHEERS is intended to guide and

capture reporting. Additional information on CHEERS 2022 can be found here.

**Title**

**1. Title**

Identify the study as an economic evaluation and specify the interventions being compared.

Page 1

**Abstract**

**2. Abstract**

Provide a structured summary that highlights context, key methods, results, and alternative analyses.

Page 3

**Introduction**

**3. Introduction: Background and Objectives**

Give the context for the study, the study question, and its practical relevance for decision making in policy or practice.

Page 4

**Methods**

**4. Health economic analysis plan**

Indicate whether a health economic analysis plan was developed and where available.

Page 5

**5. Study population**

Describe characteristics of the study population (such as age range, demographics, socioeconomic, or clinical characteristics).

Page 5

**6. Setting and location**

Provide relevant contextual information that may influence findings.

Page 5

**7. Comparators**

Describe the interventions or strategies being compared and why chosen.

Page 7

**8. Perspective**

State the perspective(s) adopted by the study and why chosen.

Page 4

**9. Time horizon**

State the time horizon for the study and why appropriate.

Page 7

**10. Discount rate**

Report the discount rate(s) and reason chosen.

Page 8

**11. Selection of outcomes**

Describe what outcomes were used as the measure(s) of benefit(s) and harm(s).

Page 7-8

**12. Measurement of outcomes**

Describe how outcomes used to capture benefit(s) and harm(s) were measured.

Page 6

**13. Valuation of outcomes**

Describe the population and methods used to measure and value outcomes.

Page 7-8

**14. Measurement and valuation of resources and costs**

Describe how costs were valued.

Page 6

**15. Currency, price date, and conversion**

Report the dates of the estimated resource quantities and unit costs, plus the currency and year of conversion.

Page 6

**16. Rationale and description of model**

If modeling is used, describe in detail and why used. Report if the model is publicly available and where it can be accessed.

Page 5

**17. Analytics and assumptions**

Describe any methods for analyzing or statistically transforming data, any extrapolation methods, and approaches for validating any model used.

Page 8

**18. Characterizing heterogeneity**

Describe any methods used for estimating how the results of the study vary for subgroups.

Page 5-6

**19. Characterizing distributional effects**

Describe how impacts are distributed across different individuals or adjustments made to reflect priority populations.

Page 5-6

**20. Characterizing uncertainty**

Describe methods to characterize any sources of uncertainty in the analysis.

Page 8

**21. Approach to engagement with patients and others affected by the study**

Describe any approaches to engage patients or service recipients, the general public, communities, or stakeholders (eg, clinicians or payers) in the design of the study.

Page 8

**Results**

**22. Study parameters**

Report all analytic inputs (eg, values, ranges, references) including uncertainty or distributional assumptions.

Appendix

**23. Summary of main results**

Report the mean values for the main categories of costs and outcomes of interest and summarize them in the most appropriate overall measure.

Page 9-11

**24. Effect of uncertainty**

Describe how uncertainty about analytic judgments, inputs, or projections affects findings. Report the effect of choice of discount rate and time horizon, if applicable.

Page 9-11

**25. Effect of engagement with patients and others affected by the study**

Report on any difference patient/service recipient, general public, community, or stakeholder involvement made to the approach or findings of the study.

Page 9-11

**Discussion**

**26. Study findings, limitations, generalizability, and current knowledge**

Report key findings, limitations, ethical, or equity considerations not captured and how these could impact patients, policy, or practice.

Page 12-14

**Other Relevant Information**

**27. Source of funding**

Describe how the study was funded and any role of the funder in the identification, design, conduct, and reporting of the analysis.

Page 15

**28. Conflicts of interest**

Report authors’ conflicts of interest according to journal or International Committee of Medical Journal Editors requirements.

Page 15
