# Supplementary material for: Optimal testing frequency for sexually transmitted infections among men who have sex with men and transgender women who use HIV pre-exposure prophylaxis in Australia, Brazil and Thailand: a cost-effectiveness analysis
Source: Lancet Reg Health West Pac. 2026 Apr 1;69:101837. doi: 10.1016/j.lanwpc.2026.101837 (PMC13084425; doi:10.1016/j.lanwpc.2026.101837)
Supplement: Supplementary Figures S5–S9 [file mmc2.pdf]

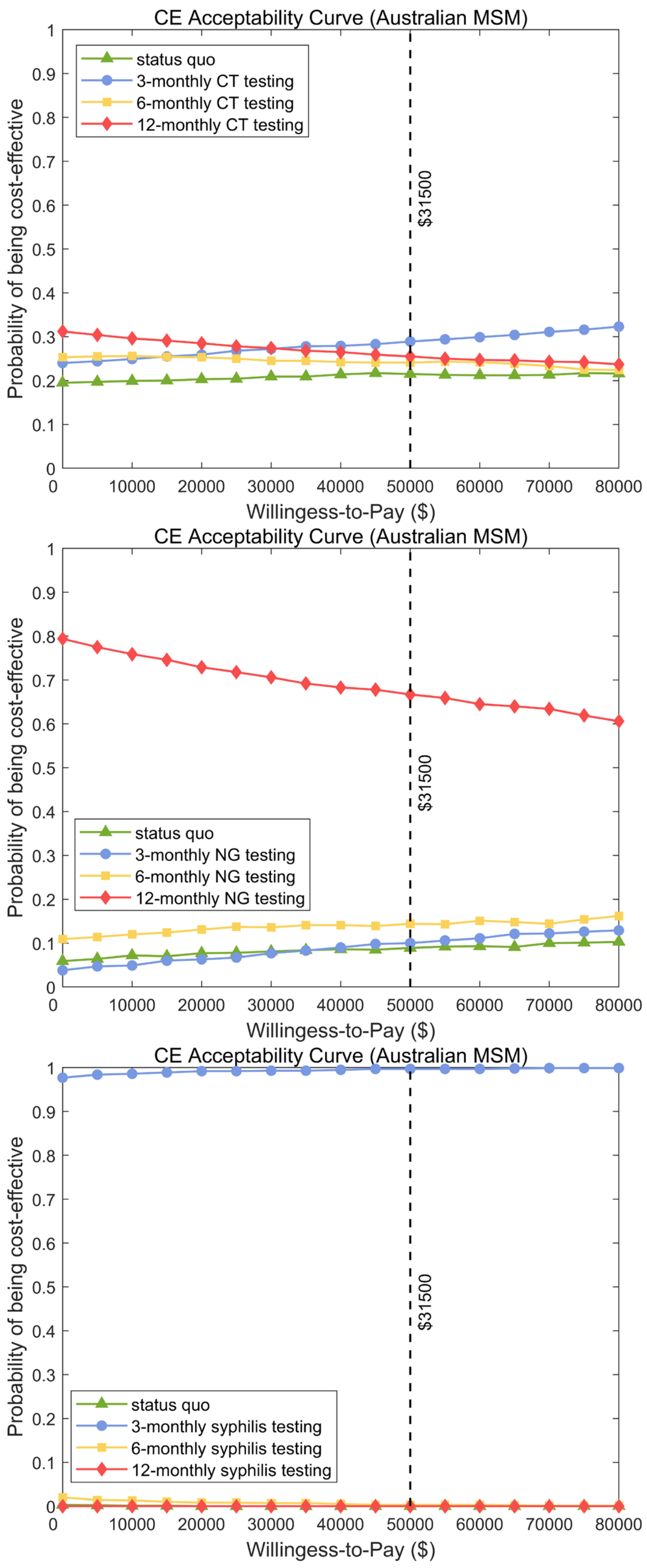

Figure S5 Probabilistic sensitivity analyses among Australian MSM

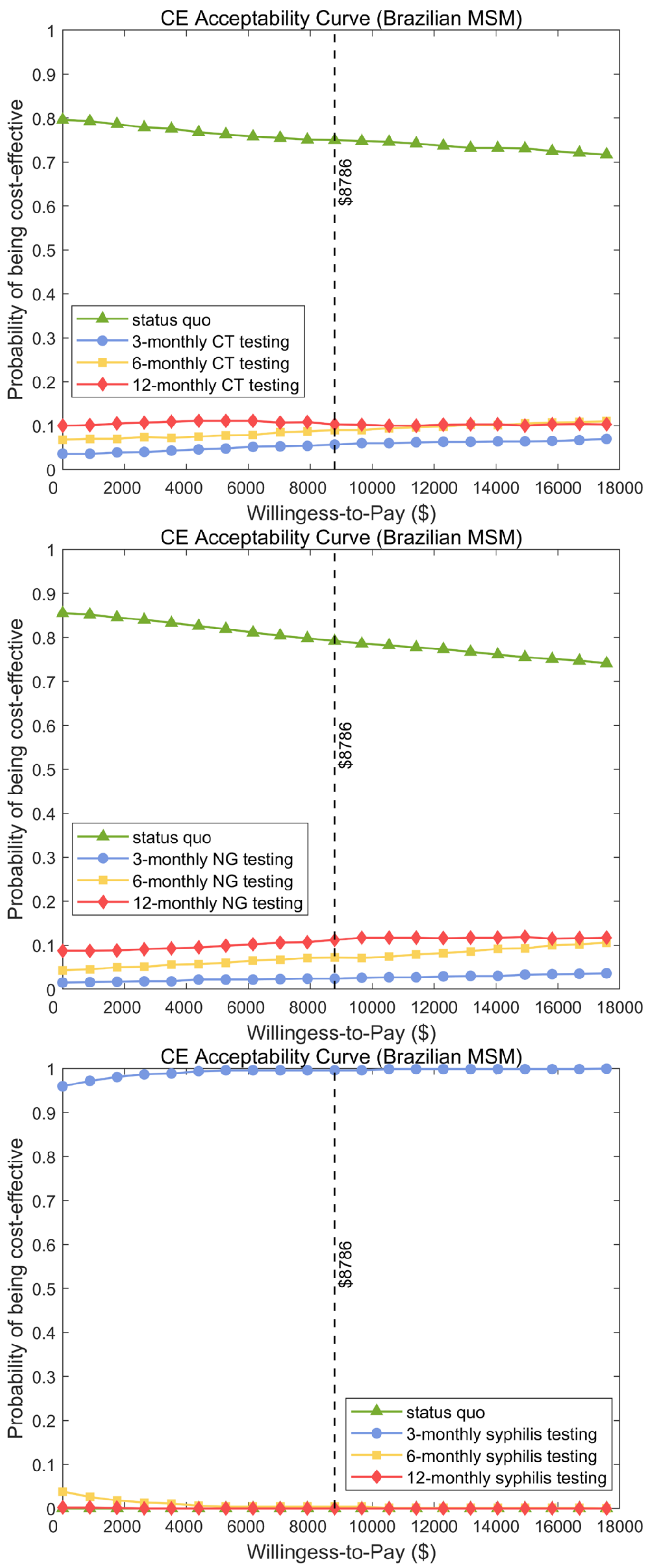

Figure S6 Probabilistic sensitivity analyses among Brazilian MSM

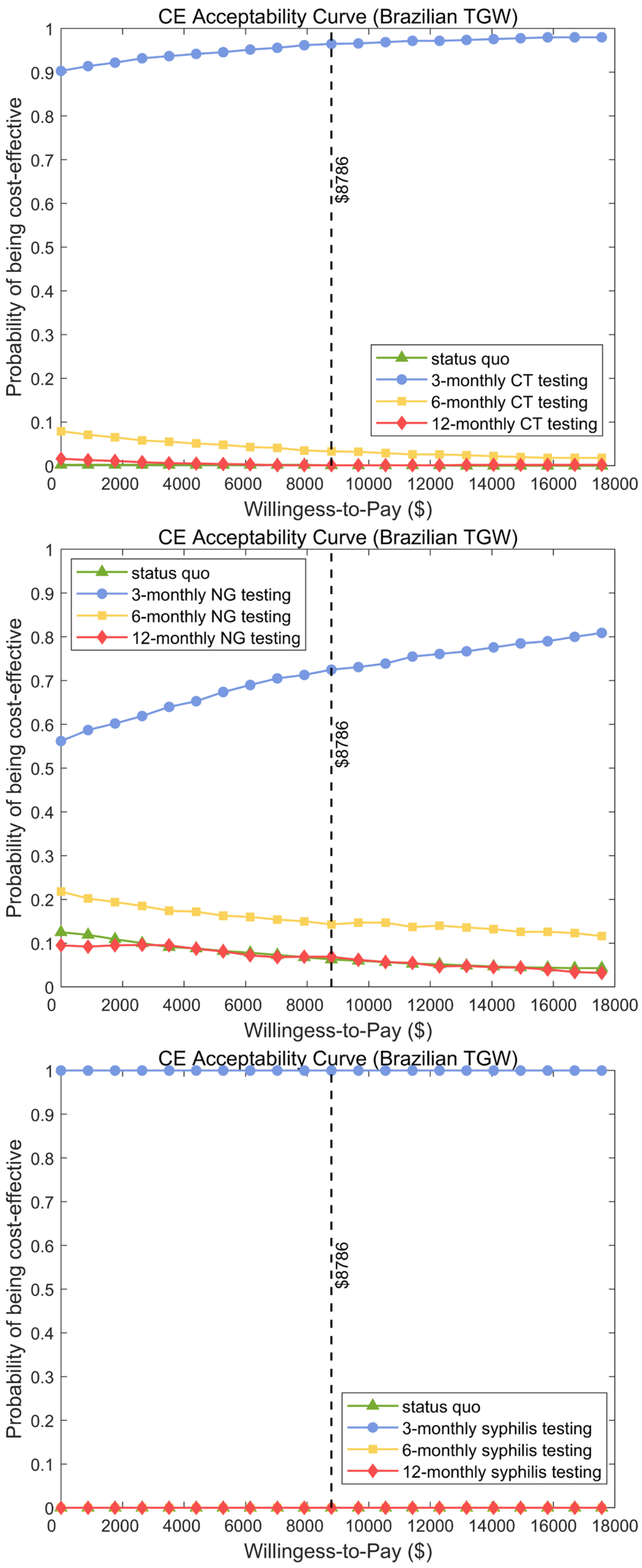

Figure S7 Probabilistic sensitivity analyses among Brazilian TGW

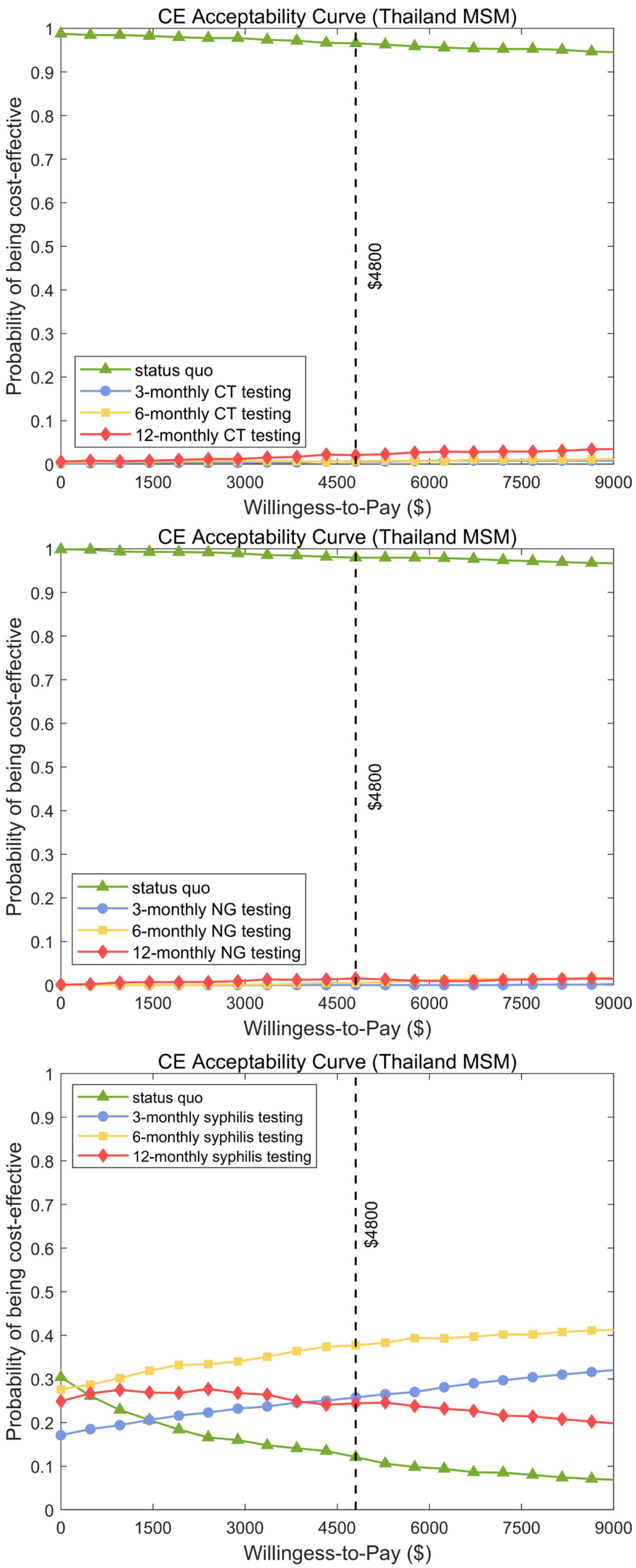

Figure S8 Probabilistic sensitivity analyses among Thailand MSM

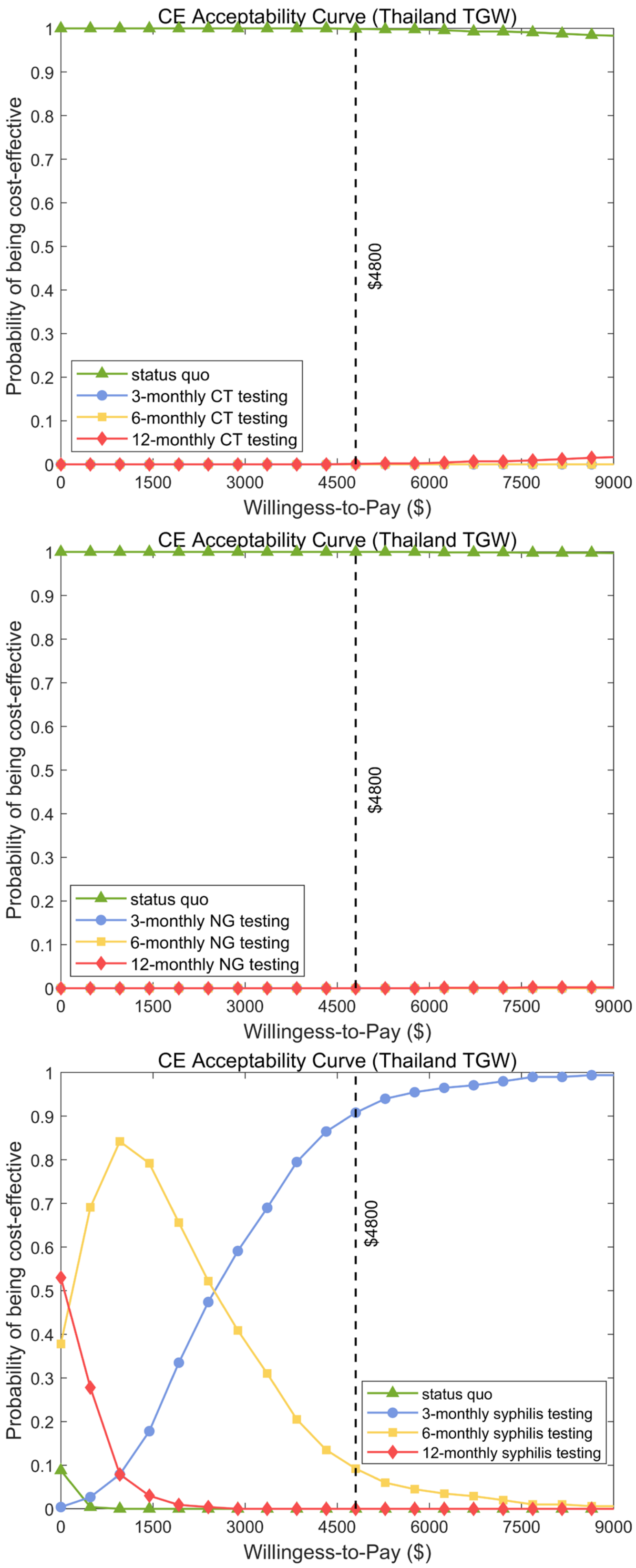

Figure S9 Probabilistic sensitivity analyses among Thailand TGW

CT=*Chlamydia trachomatis*. NG=*Neisseria gonorrhoeae.* ICER=incremental cost-effectiveness ratio. MSM=men who have sex with men. PrEP=pre-exposure prophylaxis. STI=sexually transmitted infection. TGW=transgender women. QALY=quality-adjusted life-year. WTP=willingness-to-pay.
